# Design and synthesis of phosphonium ionic liquids exhibiting strong fluorescence in various solvents and liquid or glassy state

**DOI:** 10.1016/j.jil.2025.100156

**Published:** 2025-05-14

**Authors:** David King, Yan P. Arnaiz, Hari D. Mandal, Haesook Han, Pradip K. Bhowmik

**Affiliations:** a Department of Chemistry and Biochemistry, University of Nevada Las Vegas, 4505 S. Maryland Parkway, Box 454003, Las Vegas, NV 89154, USA; b Department of Biology and Chemistry, Texas A&M International University, 5201 University Boulevard, Laredo, TX 78041, USA

**Keywords:** Synthesis, Structural design, Phosphonium ionic liquids, Dicationic ionic liquids, Glass, Fluorescence, Liquid state fluorescence

## Abstract

Liquid fluorescent ILs (LFILs) are rare materials that exhibit strong photoluminescent properties in the pure liquid state without the need for a solvent. While ILs are known for their optical properties in solution, fluorescence in the pure liquid state is typically weak or absent due to quenching phenomena. LFILs are extremely scarce in the literature, and only a few have been reported with absolute quantum yield (AQY) values to assess their fluorescence efficiency in the pure liquid state. A series of phosphonium ILs (PILs), containing fluorescent 5-(dimethylamino)-1-naphthalenesulfonate (DNS^−^) and 9,10-anthraquinone-2-sulfonate (AQS^−^), were synthesized as potential soft photoluminescent materials in solution and neat liquid state in very good to excellent yields. Additionally, mono and diphosphonium chloride ILs were prepared via an improved workup process to afford ILs with high purity (>99 %) compared to traditional methods. All fluorescent PILs possessed high thermal stabilities (*T*_d_ = 332–383 °C) as determined by thermogravimetric analysis (TGA). The differential scanning calorimetry (DSC) thermograms for the PILs-DNS and PILs-AQS revealed that all of them were ILs, with most being RTILs and the triphenylphosphonium containing PILs existing as glassy ILs (*T*_g_ = 21.13 and 30.13 °C) at room temperature. Their photoluminescent properties in the solution state were studied in various organic solvents, with the PILs-DNS possessing AQY values upwards of 0.95 in solution and [P_666,10_]DNS exhibited an impressively high AQY of 0.35 in the liquid state. The PILs-AQS did not possess strong photoluminescent properties in solution and no fluorescence in the liquid or glassy state. The PILs-DNS and PILs-AQS demonstrated excellent photostability, exhibiting no significant photobleaching. Study of these novel PILs containing anionic fluorophores is necessary to contribute towards the scarcity of LFILs and offer prospects in a wide range of fields, including chemical/physical sensing, optoelectronics, and biological imaging.

## 1. Introduction

Ionic liquids (ILs) are an important class of materials that have garnered intense interest over the past few decades in various fields, owing to their widespread applications in research and industry. Typically, they are organic compounds that possess melting points <100 °C or room temperature ILs (RTILs) that are liquid under ambient conditions. Common ILs, especially imidazolium-based ones, contain inherent fluorescent properties due to their extensive conjugation but are only weakly emissive in solution (Paul et al., 2005). ILs in which the fluorophore is not attributed to the cationic portion, which confers the physical properties of the IL, but originates from the anion instead have also been synthesized. This approach is taken to augment the fluorescence of ILs that are not strongly fluorescent, imparted by the anionic fluorophore (AF).

While there has been recent progress in designing novel ILs that exhibit strong fluorescence in solution such as imidazolium-based poly (IL)s (Pablos et al., 2021), novel imidazopyridine-based ILs (Kitaoka et al., 2024), and phosphonium ILs (PILs) containing fluorescent anionic coumarin (Che et al., 2022), very few have been synthesized to also exhibit appreciable light emission in the pure liquid or molten state. LFILs are rare in literature due to fluorescence quenching in the liquid phase. Strongly fluorescent quinolizinium ILs (Chen et al., 2011), phase-tunable benzobis(imidazolium) ILs (Boydston et al., 2007), rare-earth metal containing PILs (Alvarez Vicente et al., 2016), and tetraalkylammonium ILs incorporating fluorescein anions (Rodrigues et al., 2013) have been reported to show fluorescence in the liquid state. However, most LFILs are not reported with their absolute quantum yield (AQY) in the liquid state, which is a crucial parameter for evaluating fluorescence efficiency. Campbell et al., 2014 reported color-tunable ILs containing simple salicylate anions possessing AQY values of 0.03–0.23 in the liquid state, which were the first few LFILs with measured AQY in the liquid state. Since then, there have been no reports of LFILs with appreciable AQY values, highlighting a significant gap in the literature. Liquid fluorescent ILs (LFILs) are promising as soft luminescent materials or photofluids which find various applications in flexible optoelectronics (Mahato et al., 2023) and in the chemical sensing of gaseous analytes, small organic molecules, metals, and nitro explosives (Ilyas et al., 2024; Gan et al., 2020).

PILs have been touted as the next generation of ILs due to their extraordinary stability under high temperatures/basic conditions and versatility in applications compared to traditional tetraalkylammonium ILs (Luo et al., 2012). Since they intrinsically do not possess any chromophore or fluorophore, an AF must be incorporated for photoluminescent PILs. RT-PILs are typically purified by high temperature vacuum stripping to remove any unreacted or produced volatile byproducts (Bradaric et al., 2003). Despite this route, the highest purity reported using this technique was 97 % (Cieniecka-Rosłonkiewicz et al., 2005), which causes issues for applications that require ultrapure ILs, such as in measuring their optical properties.

For the present study, 5-(dimethylamino)-1-naphthalenesulfonate (dansyl, DNS^−^) and 9,10-anthraquinone-2-sulfonate (AQS^−^) were selected as the AFs to incorporate into the starting PILs. The dansyl moiety has been used for cellular imaging and for quantifying a wide range of analytes such as proteins, Cu(II) (Huang et al., 2014), lignins (Salanti et al., 2022), folic acid (Mukherjee et al., 2022) and even elimination of mercury (Li et al., 2023). ILs based on the DNS moiety, such as fluorescent naphthalenesulfonamide linked to imidazolium (Zhang et al., 2011) and phosphonium 8-anilinonaphthalene-1-sulfonate (Delgado et al., 2015), have also been investigated for their fluorescence in the liquid state. However, the absolute quantum yield in the pure liquid state was either not reported or was very low (1 %), respectively. NaAQS is well known for its photoredox properties and has been used as a component for the decolorization of azo dyes (Huang et al., 2022), *in situ* fluorescence probe for DNA (Li et al., 1997), electrode materials (Takimoto et al., 2022), and as a catalyst in visible-light-driven aerobic oxidative dehydrogenation of alkyl 2-phenylhydrazinecarboxylates (Tran and Kim, 2022). [P_666,14_]AQS has been prepared in the past but was only studied for its redox properties (Doherty et al., 2015).

**Scheme 1.**
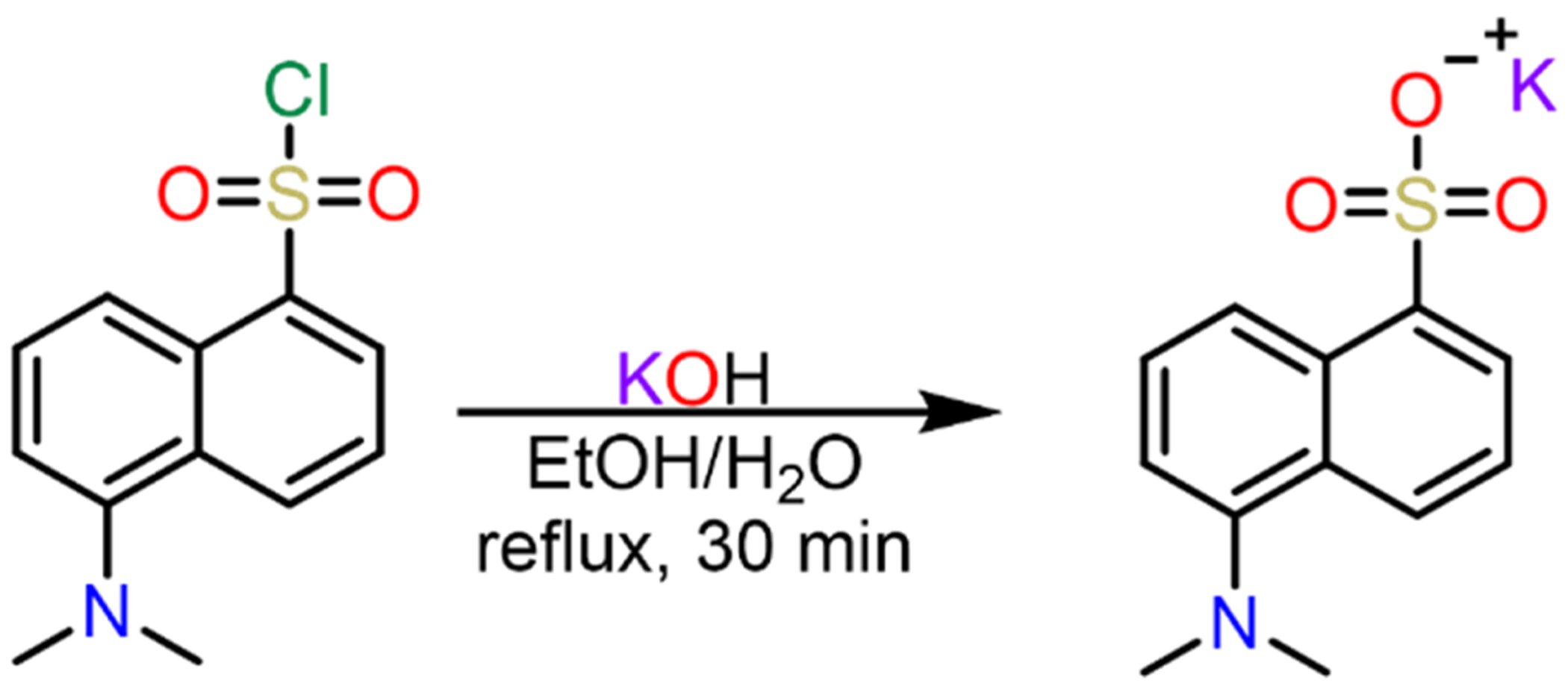
Synthesis of potassium 5-(dimethylamino)-1-naphthalenesulfonate (KDNS).

**Scheme 2.**
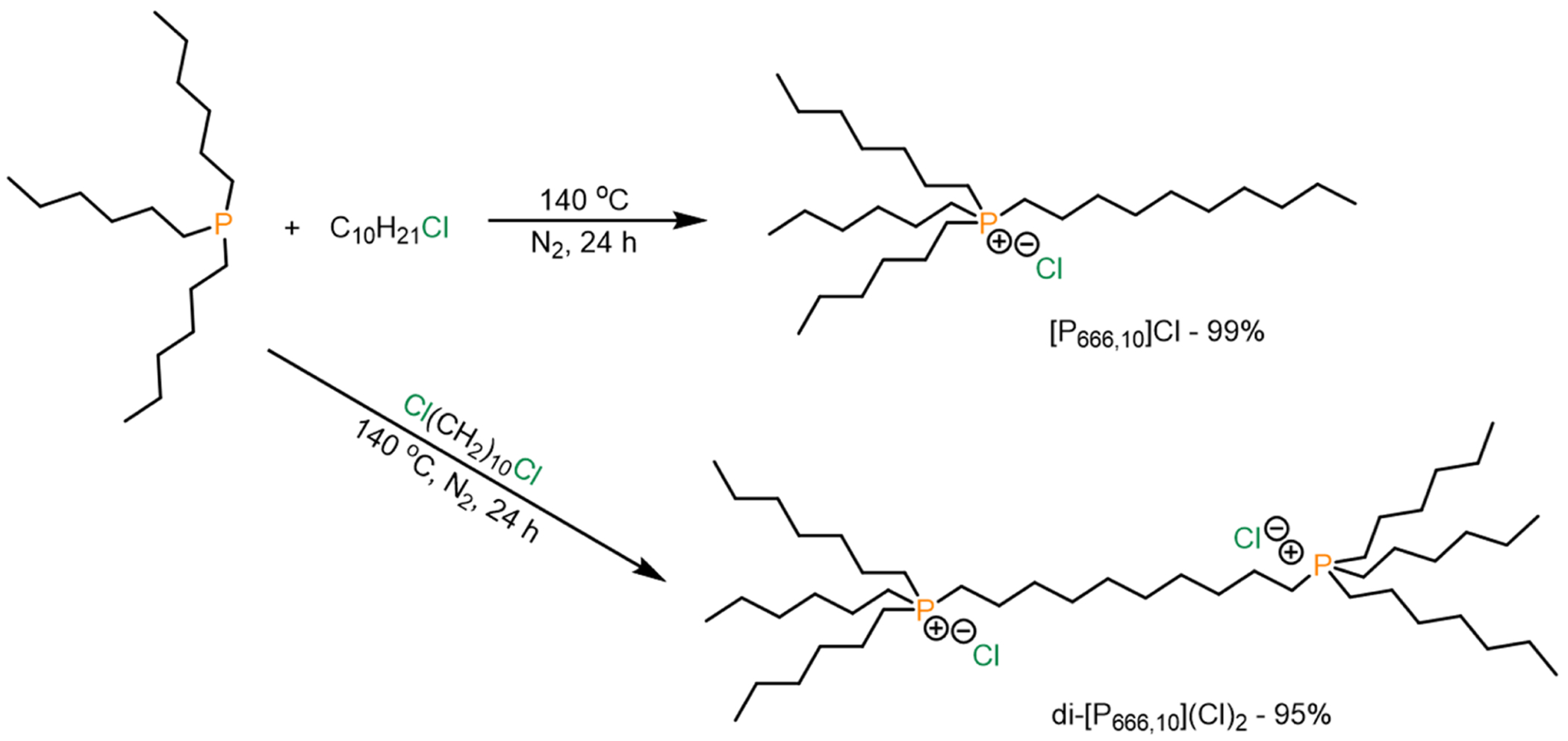
Synthesis of phosphonium chloride ILs [P_666,10_]Cl and di-[P_666,10_](Cl)_2_. [P_PhPhPh,10_]Br was synthesized according to the literature procedure (Sommers et al., 2022).

**Scheme 3.**
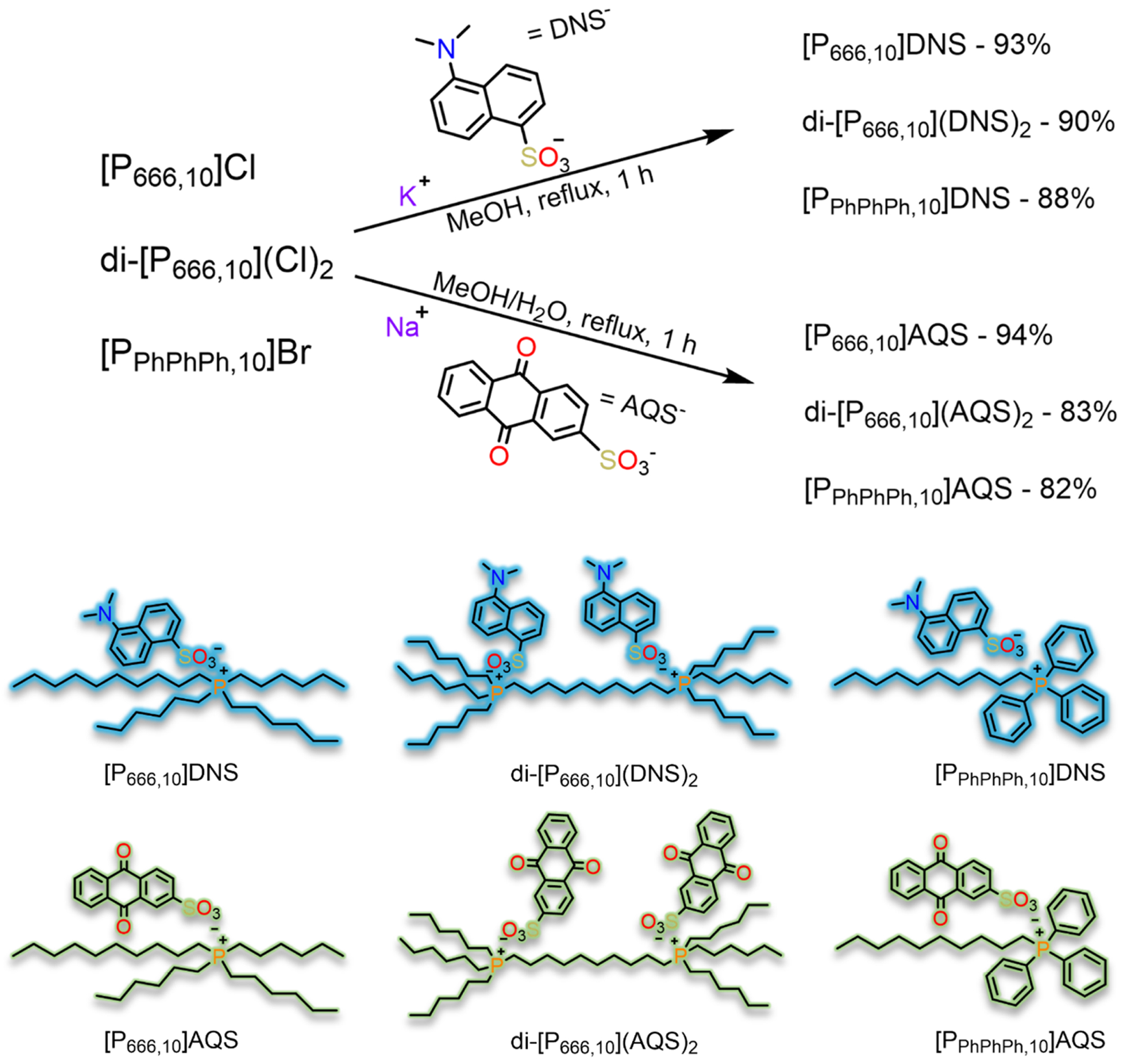
Synthesis of fluorescent phosphonium ILs-DNS and AQS.

In this work, different PILs containing fluorescent anions DNS and AQS were synthesized with a new workup approach to obtain high purity ILs. [P_666,10_]^+^, di-[P_666,10_]^2+^, and [P_PhPhPh,10_]^+^, were chosen to investigate the structural effects on melting points and photoluminescent properties. Their thermal behavior, as well as their fluorescence properties in several solvents and the pure liquid state, have been investigated herein (Schemes 1, 2, and 3).

## 2. Materials and methods

### 2.1. General information

All chemicals and solvents were reagent grade and purchased from commercial vendors (Acros Organics, Alfa-Aesar, Sigma-Aldrich, and TCI America) and were used as received. The ^1^H, ^13^C, and ^31^P nuclear magnetic resonance (NMR) salt solutions were prepared by dissolving 10 mg of each of the salts in 1 mL acetone-*d*_6_, and the spectra were recorded by using VNMR 400 spectrometer operating at 400, 100, and 162 MHz, respectively. The NMR spectra were recorded at room temperature and chemical shifts were referenced to tetramethylsilane (TMS) for ^1^H and ^13^C nuclei. Elemental analyses were performed by Atlantic Microlab Inc., Norcross, GA.

### 2.2. Synthesis

#### 2.2.1. Procedure for the synthesis of KDNS

0.294 g (5.25 mmol) of potassium hydroxide dissolved in 2.50 mL of water was added to 0.671 g of dansyl chloride (2.50 mmol) in 10 mL ethanol. The solution was brought to reflux for 30 min. Additional water was added as needed to dissolve all solids. The solvent was then removed by a rotary evaporator and the crude mixture was recrystallized from methanol to yield 0.614 g (85 %) of KDNS.

^1^H NMR (400 MHz, D_2_O) *δ*=8.43–8.41 (d, *J* = 8.8 Hz, 1H), 8.36–8.34 (d, *J* = 8.4 Hz, 1H), 8.19–8.17 (dd, *J* = 7.2, 1.2 Hz, 1H), 7.68–7.62 (m, 2H), 7.37–7.35 (d, *J* = 7.6 Hz, 1H), 2.84 (s, 6H). ^13^C NMR (100 MHz, D_2_O) *δ*=149.91, 138.46, 129.30, 128.56, 127.59, 127.56, 126.02, 124.13, 120.68, 115.38, 44.77.

#### 2.2.2. Procedure for the synthesis of phosphonium chloride ILs

Synthesis of [P_666,10_]Cl: 3.097 g (10.81 mmol) of trihexylphosphine was combined with 9.551 g (54 mmol) of 1-chlorodecane. The reaction was slowly brought to 140 °C under nitrogen atmosphere and reacted for 24 h. Nitrogen gas flow must be controlled to prevent the evaporation of starting materials. The oil was dissolved in 15 mL acetonitrile and then extracted with hexane (3 × 25 mL). Na_2_SO_4_ was added to the solution to remove any moisture, subsequently filtered, then solvent was removed by a rotary evaporator. Finally, the clear oil was dried *in vacuo* to furnish 4.930 g (99 %) of [P_666,10_]Cl.

^1^H NMR (400 MHz, acetone-*d*_6_) *δ*=2.60–2.53 (m, 8H), 1.71–1.69 (m, 8H), 1.50 (m, 8H), 1.34–1.29 (m, 24H), 0.91–0.88 (m, 12H). ^13^C NMR (100 MHz, acetone-*d*_6_) *δ*=32.62, 31.79, 31.29, 31.14, 23.32, 23.06, 22.20, 22.16, 19.64, 19.16, 14.35, 14.27. ^31^P NMR (162 MHz, acetone-*d*_6_) *δ*=34.84. Anal. Calc for C_28_H_60_PCl • H_2_O (481.23 g/mol): C, 69.89; H, 13.06 %. Found C, 70.30; H, 12.94 % (monohydrate).

Synthesis of di-[P_666,10_](Cl)_2_: 2.740 g (9.56 mmol) of trihexylphosphine was combined with 0.808 g (3.83 mmol) of 1,10-dichlorodecane. The workup procedure for [P_666,10_]Cl was followed, except that extraction with hexane was repeated 5 times instead of 3. Finally, the clear oil was dried *in vacuo* to furnish 2.838 g (95 %) of di-[P_666,10_](Cl)_2_.

^1^H NMR (400 MHz, acetone-*d*_6_) *δ*=2.71–2.55 (m, 16H), 1.73–1.69 (m, 16H), 1.52–1.48 (m, 16H), 1.35–1.34 (m, 32H), 0.91–0.88 (t, *J* = 7.2 Hz, 18H). ^13^C NMR (100 MHz, acetone-*d*_6_) *δ*=31.82, 31.32, 31.18, 23.09, 22.28, 22.24, 19.67, 19.20, 14.32. ^31^P NMR (162 MHz, acetone-*d*_6_) *δ*=34.81. Anal. Calc for C_46_H_98_P_2_Cl_2_ • H_2_O (802.15 g/mol): C, 68.88; H, 12.57 %. Found C, 69.12; H, 12.51 % (monohydrate).

#### 2.2.3. Procedure for the synthesis of phosphonium DNS ILs

Synthesis of [P_666,10_]DNS: 0.751 g (1.62 mmol) of [P_666,10_]Cl was added to a clear solution of 0.587 g (2.03 mmol) of KDNS in 30 mL of boiling methanol. After reacting for 1 hour, the solvent was removed by a rotary evaporator. The oily residue was dissolved in 10 mL of dichloromethane and extracted with water (3 × 20 mL). Na_2_SO_4_ was added to the solution, subsequently filtered, then solvent was removed by a rotary evaporator. Finally, the faintly yellow-clear oil was dried *in vacuo* to furnish 1.050 g (95 %) of [P_666,10_]DNS.

^1^H NMR (400 MHz, acetone-*d*_6_) *δ*=8.90–8.88 (d, *J* = 8.4 Hz, 1H), 8.24–8.22 (d, *J* = 8.8 Hz, 1H), 8.16–8.14 (dd, *J* = 6.8, 1.2 Hz, 1H), 7.40–7.33 (m, 2H), 7.11–7.09 (dd, *J* = 7.6 Hz, 0.8, 1H), 2.83 (s, 6H), 2.44–2.37 (m, 8H), 1.68–1.62 (m, 8H), 1.45–1.42 (m, 8H), 1.30–1.28 (m, 24H), 0.89–0.85 (m,12H). ^13^C NMR (100 MHz, acetone-*d*_6_) *δ*=151.50, 146.82, 132.31, 130.44, 125.80, 125.48, 124.71, 124.17, 114.50, 45.69, 32.62, 31.75, 31.18, 31.03, 23.33, 23.04, 22.10, 22.06, 19.39, 18.91, 14.36, 14.27. ^31^P NMR (162 MHz, acetone-*d*_6_) *δ*=34.73. Anal. Calc for C_40_H_72_NPO_3_S (678.05 g/mol): C, 70.86; H, 10.70; N, 2.07; S, 4.73 %. Found C, 70.27; H, 11.01; N, 1.93; S, 4.20 %.

Synthesis of di-[P_666,10_](DNS)_2_: 0.668 g (0.852 mmol) of di-[P_666,10_](Cl)_2_ was dissolved in minimum methanol and added to a clear solution of 0.616 g (2.13 mmol) KDNS in 30 mL of boiling methanol. The procedure for [P_666,10_]DNS was followed for workup. Finally, the faint yellow-clear oil was dried *in vacuo* to furnish 0.931 g (90 %) of di-[P_666,10_](DNS)_2_.

^1^H NMR (400 MHz, acetone-*d*_6_) *δ*=8.91–8.88 (d, *J* = 8.8 Hz, 2H), 8.27–8.25 (d, *J* = 8.4 Hz, 2H), 8.18–8.16 (dd, *J* = 6.8, 1.2 Hz, 2H), 7.42–7.36 (m, 4H), 7.23–7.11 (d, *J* = 7.2 Hz, 2H), 2.85 (s, 12H), 2.47–2.30 (m, 16H), 1.70–1.54 (m, 16H), 1.48–1.25 (m, 48H), 0.88–0.84 (t, *J* = 6.8 Hz, 18H). ^13^C NMR (100 MHz, acetone-*d*_6_) *δ*=151.56, 146.84, 132.32, 130.48, 125.87, 125.84, 125.51, 124.72, 124.21, 114.55, 45.72, 31.75, 31.17, 31.01, 23.05, 22.09, 22.05, 19.32, 18.85, 14.30. ^31^P NMR (162 MHz, acetone-*d*_6_) *δ*=34.83. Anal. Calc for C_70_H_122_N_2_P_2_O_6_S_2_ (1213.82 g/mol): C, 69.27; H, 10.13; N, 2.31; S, 5.28 %. Found C, 69.04; H, 10.31; N, 2.23; S, 5.07 %.

Synthesis of [P_PhPhPh,10_]DNS: 2.50 g (5.17 mmol) of [P_PhPhPh,10_]Br was dissolved in minimum methanol and added to a clear solution of 1.871 g (6.47 mmol) KDNS in 60 mL of boiling methanol. The procedure for [P_666,10_]DNS was followed for workup. Finally, the desired compound was dried *in vacuo* to furnish 2.97 g (88 %) of glassy yellow [P_PhPhPh,10_]DNS.

^1^H NMR (400 MHz, acetone-*d*_6_) *δ*=8.92–8.90 (d, *J* = 8.8 Hz, 1H), 8.22–8.20 (d, *J* = 8.4 Hz, 1H), 8.18–8.16 (dd, *J* = 6.8, 1.2 Hz, 1H), 7.97–7.88 (m, 9H), 7.79–7.74 (m, 6H), 7.35–7.28 (m, 2H), 7.08–7.07 (d, *J* = 7.2 Hz, 1H), 3.67–3.60 (m, 2H), 2.82 (s, 6H), 1.73–1.67 (m, 2H), 1.57–1.49 (m, 2H), 1.28–1.21 (m, 12H), 0.88–0.84 (t, *J* = 6.8 Hz, 3H). ^13^C NMR (100 MHz, acetone-*d*_6_) *δ*=151.44, 146.99, 135.80, 135.77, 134.77, 134.67, 132.39, 131.24, 131.12, 130.41, 125.69, 125.62, 125.52, 124.92, 124.22, 120.24, 119.39, 114.44, 45.70, 32.59, 31.14, 30.97, 23.31, 23.16, 23.11, 22.29, 21.79, 14.36. ^31^P NMR (162 MHz, acetone-*d*_6_) *δ*=25.63. Anal. Calc for C_40_H_48_NPO_3_S (653.83 g/mol): C, 73.48; H, 7.40; N, 2.14; S, 4.90 %. Found C, 73.22; H, 7.34; N, 2.14; S, 4.74 %.

Synthesis of [P_666,10_]AQS: 0.695 g (1.50 mmol) of [P_666,10_]Cl was combined with 0.583 g (1.88 mmol) of NaAQS in 20 mL of hot methanol. 5 mL of water was added until the solution became clear. After refluxing for 1 hour, the solution was cooled, and water was added until an oil separated. The oil was transferred and dissolved in 10 mL of dichloromethane and extracted with water (3 × 20 mL). Na_2_SO_4_ was added to the solution, subsequently filtered, then solvent was removed by a rotary evaporator. The final faintly yellow-clear oil was dried *in vacuo* to furnish 1.004 g (94 %) of [P_666,10_]AQS.

^1^H NMR (400 MHz, acetone-*d*_6_) *δ*=8.72 (s, 1H), 8.30–8.29 (m, 2H), 8.26 (s, 2H), 7.95–7.93 (m, 2H), 2.48–2.40 (m, 8H), 1.71–1.65 (m, 8H), 1.49–1.45 (m, 8H), 1.31–1.23 (m, 24H), 0.87–0.84 (t, *J* = 7.2 Hz, 12H). ^13^C NMR (100 MHz, acetone-*d*_6_) *δ*=183.38, 183.15, 156.38, 135.16, 135.13, 134.60, 134.51, 134.15, 133.83, 132.58, 127.74, 127.72, 127.60, 125.45, 32.61, 31.77, 31.55, 31.40, 31.26, 31.10, 23.32, 23.06, 22.11, 22.07, 19.44, 18.97, 14.36, 14.26. ^31^P NMR (162 MHz, acetone-*d*_6_) *δ*=34.96. Calc for C_42_H_67_O_5_PS (715.03 g/mol): C, 70.55; H, 9.45; S, 4.48 %. Found C, 70.33; H, 9.59; S, 4.29 %.

Synthesis of di-[P_666,10_](AQS)_2_: 0.823 g (1.05 mmol) of [P_666,10_](Cl)_2_ was combined with 0.814 g (2.62 mmol) of NaAQS in 20 mL of hot methanol + 5 mL of water. The procedure for [P_666,10_]AQS was followed for workup. Finally, the beige solid was dried *in vacuo* to furnish 1.116 g (83 %) of di-[P_666,10_](AQS)_2_.

^1^H NMR (400 MHz, acetone-*d*_6_) *δ*=8.71 (s, 2H), 8.29–8.26 (m, 8H), 7.96–7.94 (m, 4H), 2.44–2.37 (m, 16H), 1.66–1.64 (m, 16H), 1.43 (m, 16H), 1.43 (m, 32H), 0.83 (m, 18H). ^13^C NMR (100 MHz, acetone-*d*_6_) *δ*=183.34, 183.09, 156.20, 135.24, 134.51, 134.42, 134.14, 133.84, 132.54, 127.74, 127.65, 125.42, 31.76, 31.25, 31.10, 23.06, 22.11, 22.06, 19.37, 18.90, 14.27. ^31^P NMR (162 MHz, acetone-*d*_6_) *δ*=34.89. Anal. Calc for C_74_H_112_O_10_P_2_S_2_ (1287.77 g/mol): C, 69.02; H, 8.77; S, 4.98 %. Found C, 69.30; H, 8.93; S, 4.84 %.

Synthesis of [P_PhPhPh,10_]AQS: 1.343 g (2.77 mmol) of [P_PhPhPh,10_]Br was combined with 1.077 g (3.47 mmol) of NaAQS in 35 mL of hot methanol + 10 mL of water. The procedure for [P_666,10_]AQS was followed for workup. Finally, the desired compound was dried *in vacuo* to furnish 1.569 g (82 %) of glassy yellow [P_PhPhPh,10_]AQS.

^1^H NMR (400 MHz, acetone-*d*_6_) *δ*=8.71 (s, 1H), 8.28–8.27 (m, 2H), 8.20 (s, 2H), 7.98–7.91 (m, 11H), 7.81–7.79 (m, 6H), 3.68–3.61 (m, 2H), 1.73–1.70 (m, 2H), 1.58–1.56 (m, 2H), 1.28–1.17 (m, 12H), 0.85–0.81 (t, *J* = 6.8 Hz, 3H). ^13^C NMR (100 MHz, acetone-*d*_6_) *δ*=183.37, 183.15, 156.41, 135.84, 135.08, 134.75, 134.65, 134.47, 134.07, 133.72, 132.55, 131.26, 131.14, 127.71, 127.67, 127.50, 125.48, 120.19, 119.33, 32.55, 31.21, 31.05, 23.27, 23.16, 23.12, 22.33, 21.83, 14.33. ^31^P NMR (162 MHz, acetone-*d*_6_) *δ*=25.59. Anal. Calc for C_42_H_43_O_5_PS (690.83 g/mol): C, 73.02; H, 6.27; S, 4.64 %. Found C, 72.99; H, 6.29; S, 4.63 %.

### 2.3. Characterization techniques of fluorescent ILs

The thermal stability properties of the starting salts and ILs were assessed using a TGA Q50 instrument at a heating rate of 10 °C·min^−1^ in nitrogen. The glass and melting transition temperatures were acquired using a TA module DSC Q200 series in nitrogen, at heating and cooling rates of 10 °C·min^−1^. The temperature axis of the DSC thermograms were calibrated with reference standards of high purity indium and tin. The UV–Vis absorption spectra of fluorescent salts and ILs in spectrophotometric grade solvents (methanol, ethanol, toluene, dichloromethane, ethyl acetate, tetrahydrofuran, acetonitrile, and water) were recorded by using the absorbance module attached to PerkinElmer Fluorescence Spectrometer FL 6500. The fluorescence and excitation spectra with the use of the single-cell holder accessory, as well as solution state absolute quantum yields with the use of an integrating sphere accessory, were measured with the same spectrometer. Absolute quantum yields of the ILs in the pure liquid or glassy state were measured with a Horiba Fluorolog fluorimeter (HORIBA Instruments Inc.) also equipped with an integrating sphere.

## 3. Results and discussion

### 3.1. Synthesis of KDNS and fluorescent PILs

The synthesis of KDNS and PILs containing the fluorescent DNS or AQS counteranion was successfully achieved in very good to excellent yields. A simple hydrolysis of dansyl chloride was performed in an ethanol/water mixture using potassium hydroxide as the base. Synthesis of the phosphonium chloride ILs [P_666,10_]Cl and di-[P_666,10_](Cl)_2_ were carried out in a similar fashion via an S^2^_N_ reaction with no solvent under nitrogen in exceptional yields, which is a method traditionally employed for PIL synthesis. The controlled flow of nitrogen gas was imperative to ensure that neither the trihexylphosphine nor alkyl halide was carried out of the reaction mixture. The resulting ILs were reacted with the respective AF salt via counterion metathesis to furnish the fluorescent PILs-DNS and PILs-AQS. The purity of all synthesized ILs were determined by ^1^H, ^13^C, and ^31^P NMR spectra as provided in the supplementary material (Figures S1–S9) and elemental analysis.

An important advantage of the synthesis of [P_666,10_]Cl and di-[P_666,10_](Cl)_2_ is the modified workup process compared to traditional methods reported in the literature. Purification of RTILs can pose a challenge as recrystallization is not possible and they cannot be efficiently washed with solvents compared to solid compounds. Due to the higher tendency of PILs with longer alkyl chains to be RTILs, vacuum stripping at elevated temperatures is often employed to remove volatile impurities, yielding purer PILs. However, a major limitation in this method is that it does not remove all reaction byproducts, specifically protonated phosphonium salt and alkene isomers formed via elimination side reactions. An early study for the industrial preparation of PILs also reported hydrochloric acid as a side impurity, which can be detrimental to the potential application of these materials (Bradaric et al., 2003). The purity of trihexyl(tetradecyl)phosphonium chloride reported by Bradaric et al., 2003 was 94 % and 97 % reported by Cieniecka-Rosłonkiewicz et al., 2005, which mentioned that vacuum stripping took place at 180 °C. Vacuum stripping can be replaced by acetonitrile/hexane extraction in which the PIL is partitioned in the polar acetonitrile layer and the impurities are in the non-polar hexane layer. This method provides highly pure (>99 %) PILs for applications that require intensive care so that the impurities do not interfere with chemical processing and research in a wide variety of fields, including in the endeavor to study the optical properties of PILs synthesized with AFs.

### 3.2. Thermal stability of salts and PILs

**Fig. 1.**
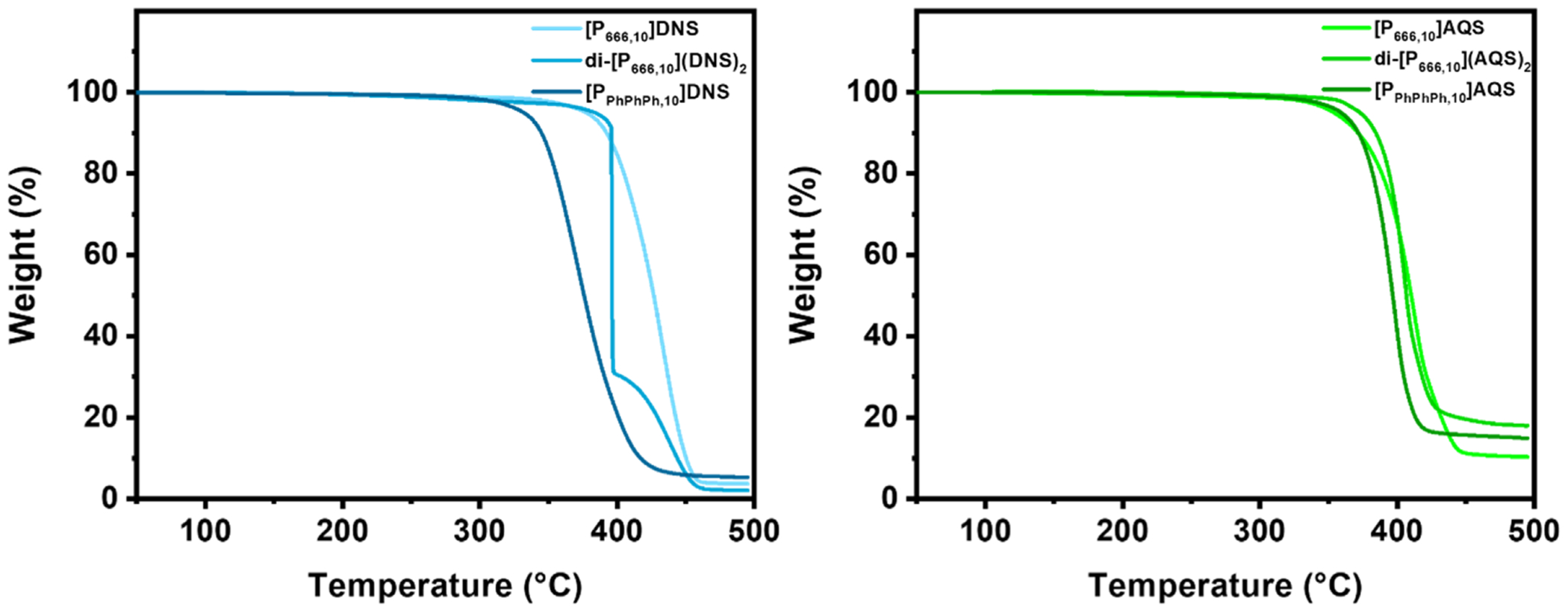
TGA thermograms of [P_666,10_], di-[P_666,10_], and [P_PhPhPh,10_] DNS (left) and AQS (right) ILs obtained at a heating rate of 10 °C/min under nitrogen. TGA thermograms of all other compounds are contained in the supplementary information (Figures S10 and S11).

The thermal stability for the starting salts and PILs were assessed by thermogravimetric analysis (TGA) and is defined as the temperature (°C) at which a 5 % weight loss occurred at a heating rate of 10 °C/min in nitrogen (Fig. 1). The *T*_d_ for KDNS and NaAQS was 368 °C and 428 °C, respectively. All PILs possessed extraordinary thermal stabilities, with *T*_d_ ranging from 332–383 °C for the PILs-DNS and 354–373 °C for PILs-AQS (Table 1). The dicationic PILs possessed the highest thermal stabilities, up to 383 °C for di-[P_666,10_](DNS)_2_, which is expected as dicationic compounds typically possess greater stability due to two charge centers that strengthen ionic interactions (Shirota et al., 2011). [P_666,14_]Cl, which has been a widely studied PIL in the past few decades, exhibits its *T*_d_ at around 320 °C compared to 301 °C for [P_666,10_]Cl (Deferm et al., 2018). [P_444,12_] and [P_444,18_] synthesized with a 1,2,3-triazolide counteranion only have a negligible difference in *T*_d_ and even *T*_g_ (Fillion et al., 2016). The non-nucleophilicity and stability of the counteranion of the PIL is the major contributor towards thermal stability, which is an important consideration in the design of these PILs. The PILs-DNS were generally more stable than the PILs-AQS with the exception of [P_PhPhPh,10_], where [P_PhPhPh,10_]AQS had a *T*_d_ 27 °C higher than [P_PhPhPh,10_]DNS, which could be due to stronger π–π interactions between the phenyl rings of the triphenyldecylphosphonium cation and AQS anion. DNS and AQS counteranions impart exceptional stability due to their weak nucleophilicity and are even comparable to [P_666,14_] synthesized with benzoate and salicylate counteranions (Khajeh et al., 2022), bio-inspired amino acid anions (Huang et al., 2022), and [P_666,14_] paired with the famously stable triflimide (Tf_2_N^−^) anion, which has a reported *T*_d_ of 390°C (Fillion et al., 2016).

### 3.3. Thermal properties of salts and PILs

**Fig. 2.**
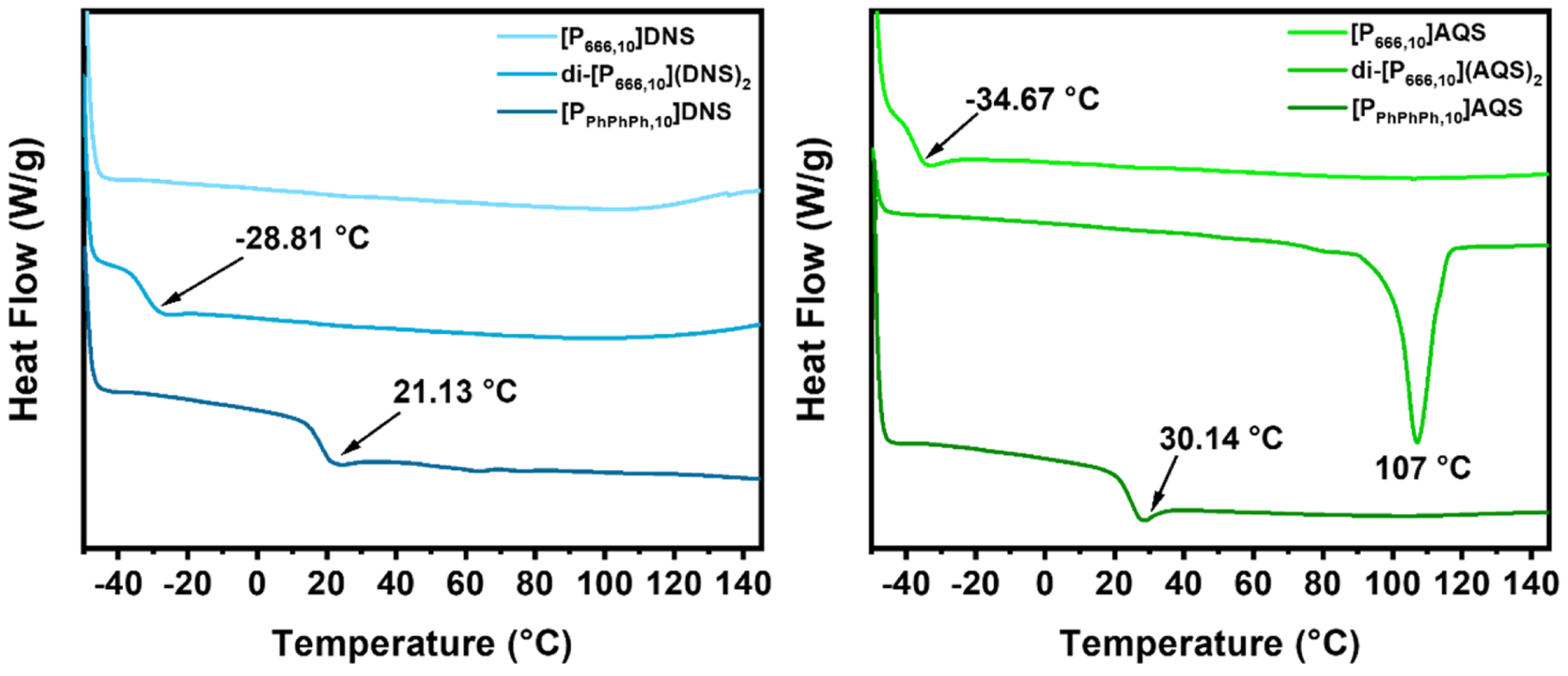
DSC thermograms of first heating cycles of [P_666,10_], di-[P_666,10_], and [P_PhPhPh,10_]DNS (left) and AQS (right) ILs obtained at a heating rate of 10 °C/min in nitrogen. Complete heating and cooling cycles for all compounds are contained in supplementary information (Figures S12-S21).

DSC thermograms of the PILs and organic salts used to synthesize them (KDNS and NaAQS) were obtained to determine if they are ILs (Fig. 2). KDNS possessed a melting point of 334 °C and NaAQS decomposed before an observable melting point. [P_666,10_]Cl and [P_666,10_]DNS did not have an observable *T*_g_ when cooled to −50 °C, but other PILs such as [P_666,14_]Cl and [P_666,14_]Tf_2_N exhibited *T*_g_ values of −75 °C and −77 °C respectively (Yao et al., 2023). Notably, variable alkyl chain lengths do not greatly affect thermal properties of PILs bearing the same counteranions, such as [P_666,n_]Tf_2_N from *n* = 2–12 (Yao et al., 2023) and phosphonium docusate ILs (Shirota et al., 2024). On the other hand, [P_666,10_]AQS exhibited *T*_g_ at −34.67 °C, comparable to other AQS based ILs (Doherty et al., 2015,). A small melting transition was observed at 40 °C for di-[P_666,10_](Cl)_2_. While di-[P_666,10_](DNS)_2_ displayed its *T*_g_ at −28.81 °C, di-[P_666,10_](AQS)_2_ had a dramatically high *T*_m_ at 107 °C. This was an unexpected compared to the phase transitions of the other PILs studied; however, the data agrees with the fact that dicationic ILs generally have higher *T*_m_ values due to stronger ionic interactions and potential symmetry depending on the DIL studied (King et al., 2024). This phenomenon could be explained by the high degree of crystallinity of NaAQS because of its exceedingly high melting point (decomposition) and the symmetry present in the PIL, which encourages stronger π–π interactions between the two AQS counteranions of dicationic di-[P_666,10_](AQS)_2_ (Anderson et al., 2005). Di-[P_666,10_](AQS)_2_ was also observed to have *T*_g_ at −0.88 °C on second heating and interestingly, no crystallization exotherm on either cooling cycle (Figure S20).

[P_PhPhPh,10_]DNS and [P_PhPhPh,10_]AQS displayed a *T*_g_ of 21.13 °C and 30.14 °C, respectively, which is expected due to the rigidity of the triphenylphosphonium (TPP) moiety and π–π interactions between the aromatic rings of the cation and anion. O’Rourke et al., 2021 reported that butyltriphenylphosphonium salts and ILs prepared with different perfluoroalkyl containing anions related to the bistriflimide anion influenced the *T*_g_ and *T*_m_ of the resulting materials depending on the perfluoroalkyl groups and the flexibility of the differing anions. In contrast to TPP tosylate salts, which exhibit considerably high *T*_m_ values (>100 °C) relative to the TPP ILs studied here, carbon chain length (*n* = 2, 3, 4, 5, 8) does not drastically affect *T*_m_ compared to the change in counteranion. This observation confirms that anions play the dominant role in affecting the thermal properties of both trialkyl and TPP ILs (Bonnet and Kariuki, 2006). Both [P_PhPhPh,10_]DNS and [P_PhPhPh,10_]AQS did not have *T*_m_ transitions, rendering them room-temperature glassy materials. All synthesized mono and diphosphonium salts were found to be ILs according to DSC measurements, despite the extraordinarily high *T*_m_ values of the starting salts (KDNS and NaAQS). PILs are known for their capacity to become amorphous materials that can exhibit unique properties known as liquid-liquid transitions, partly owing to their tendency not to undergo crystallization (Harris et al., 2021; Wojnarowska et al., 2022).

### 3.4. Optical properties of salts and PILs in solutions

The absorption properties of KDNS and the PILs-DNS were studied in ethanol, methanol, dichloromethane, acetonitrile, tetrahydrofuran, ethyl acetate, and toluene at 1–5 × 10^−5^ M concentrations. KDNS was only studied in ethanol, methanol, acetonitrile, and water due to solubility limitations. The solubility of KDNS was higher in methanol, ethanol, and water (1.0 × 10^−3^ M) than in acetonitrile, where it was only soluble up to 1.0 × 10^−4^ M. It is important to note that pairing the DNS counteranion with the phosphonium cations dramatically improves solubility, allowing the study of photoluminescent properties in a wide range of solvents. None of the PILs-DNS were soluble in water at 1.0 × 10^−4^ M due to the hydrophobicity of the trialkyl/triphenylphosphonium cation. All of their molar absorptivities were also calculated from Beer Lambert’s plots and shown in Table 2. In general, the ILs retained the intrinsic optical and photoluminescent properties of the DNS anion effectively. The UV–Visible spectra for KDNS, [P_666,10_]DNS, and di-[P_666,10_](DNS)_2_ consistently displayed three absorbance peaks each at around λabs = 217–220 nm, 245–246 nm, and 318–325 nm in various organic solvents. KDNS in water had absorbance peaks at λ_abs_ = 215, 241, and 314 nm, which are slight hypsochromic shifts compared to those in other organic solvents. Interestingly, bathochromic and hypsochromic shifts were observed for [P_666,10_]DNS in THF, where its λ_abs_ peaks were located at 237 and 306 nm in the absorption spectrum. The intermediate polarity of THF partially stabilizes the excited states of the PILs-DNS through dipole-dipole interactions, which explains the observed shifts in λ_abs_ values. [P_PhPhPh,10_]DNS had consistently displayed two peaks in various organic solvents, each at λabs = 217–220 and 313–322. Bathochromic and hypsochromic shifts were also observed for [P_PhPhPh,10_]DNS in THF, where its λ_abs_ peaks were located at 231 and 311 nm in the absorption spectrum. All UV–visible spectra are contained in the supplementary materials (Figures S22–S46).

**Fig. 3.**
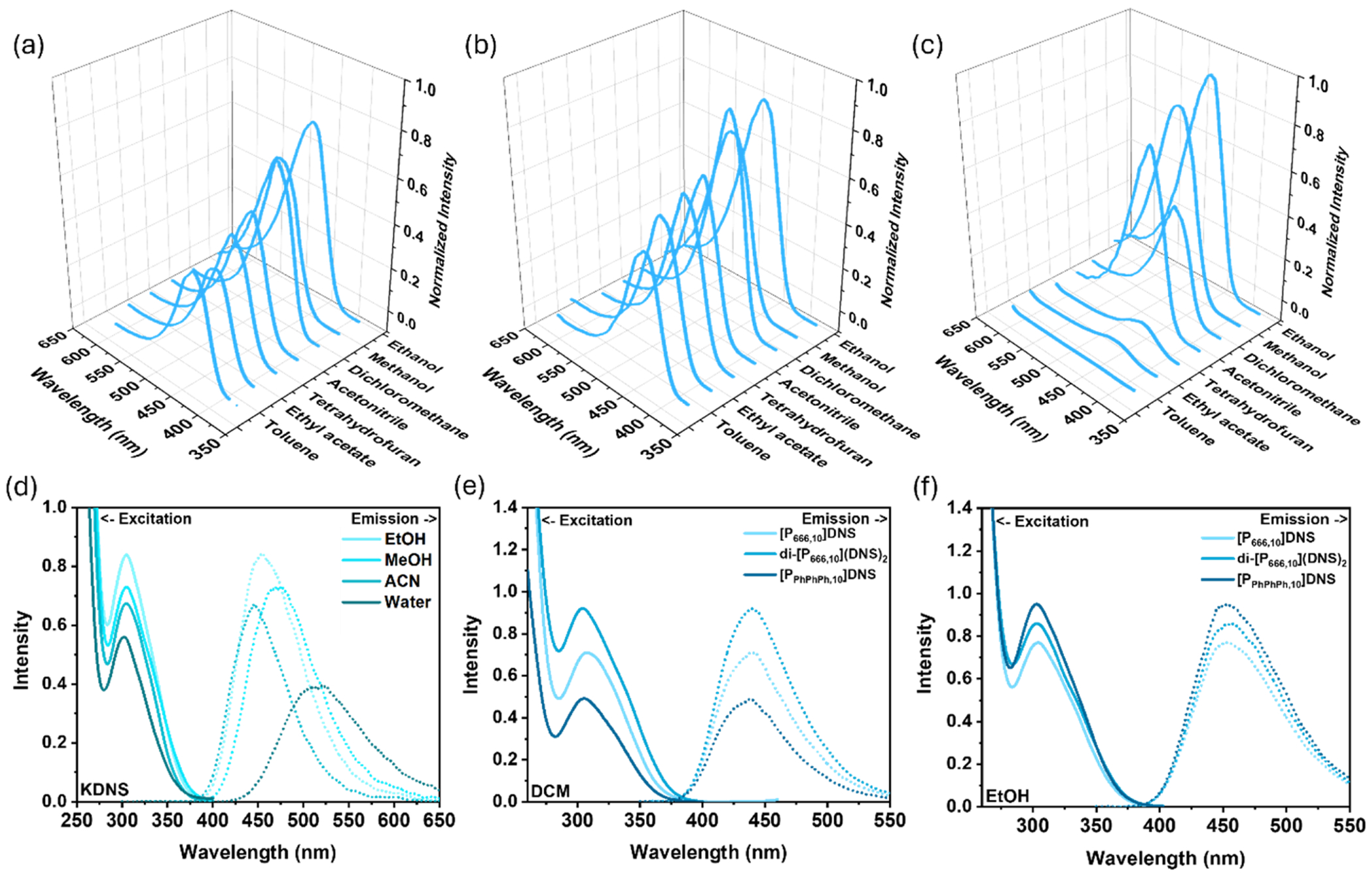
Room-temperature emission spectra (top) of (a) [P_666,10_]DNS, (b) di-[P_666,10_](DNS)_2_, and (c) [P_PhPhPh,10_]DNS in various solvents and emission/excitation spectra of (d) KDNS in various solvents, (e) PILs-DNS in DCM, and (f) PILs-DNS in EtOH. All spectra were measured at 5.0 × 10^−5^ M concentrations.

The photoluminescent properties of KDNS and PILs-DNS were studied at 5 × 10^–5^ M concentrations in their respective solvents and all spectra are contained in Fig. 3. Among the solvents studied, MeOH caused the most bathochromic shift (λ_em_ = 470–474 nm), while toluene caused the most hypsochromic shift (λ_em_ = 420–427 nm). The only exception to this is THF, where the emission peak for [P_PhPhPh,10_]DNS is observed at λ_em_ = 413 nm compared to λ_em_ = 441 nm for [P_666,10_]DNS and λ_em_ = 439 nm for di-[P_666,10_](DNS)_2_ in the same solvent. All other emission values were consistently in between, with EtOH: λ_em_ = 453–455, DCM: λem = 438–442 nm, ACN: λ_em_ = 442–445 nm, and EtOAc: λ_em_ = 428–438 nm. KDNS exhibited an emission peak at λ_em_ = 510 nm in pure water. Their AQY values were also measured in the respective solvents. KDNS in water had a AQY of 0.56, which is considerably high compared to dansyl derivatives measured in water (Li et al., 1975). The relative QY of DNS was measured in water by Chen, 1966, utilizing quinine in 1 N sulfuric acid as a standard and was reported to be 0.37. The AQY values of KDNS and the PILs-DNS were highest in polar protic solvents, MeOH and EtOH, ranging from 0.64 to 0.84 and 0.77–0.95, respectively. The highest AQY was observed for [P_PhPhPh,10_]DNS in EtOH, reaching an impressive value of 0.95. Di-[P_666,10_](DNS)_2_ performed the best in DCM, with an AQY of 0.91. All other AQY values in DCM, ACN, THF, EtOAc, and toluene were in the range of 0.42–0.71, with the exception of [P_PhPhPh,10_]DNS in THF, EtOAc, and toluene. The fluorescence of [P_PhPhPh,10_]DNS was drastically quenched in these solvents, with quantum yield values of 0.09 for THF, and 0.05 for EtOAc and toluene, suggesting that these solvents do not properly stabilize the charge transfer state between the DNS anion and TPP moiety. TPP is well-known for its capacity to target mitochondria and pharmacophores are typically conjugated to the alkyl chain as cargo to deliver into the organelle as a drug therapy (Zielonka et al., 2017), making [P_PhPhPh,10_]DNS a promising candidate for mitochondrial-imaging strategies.

**Fig. 4.**
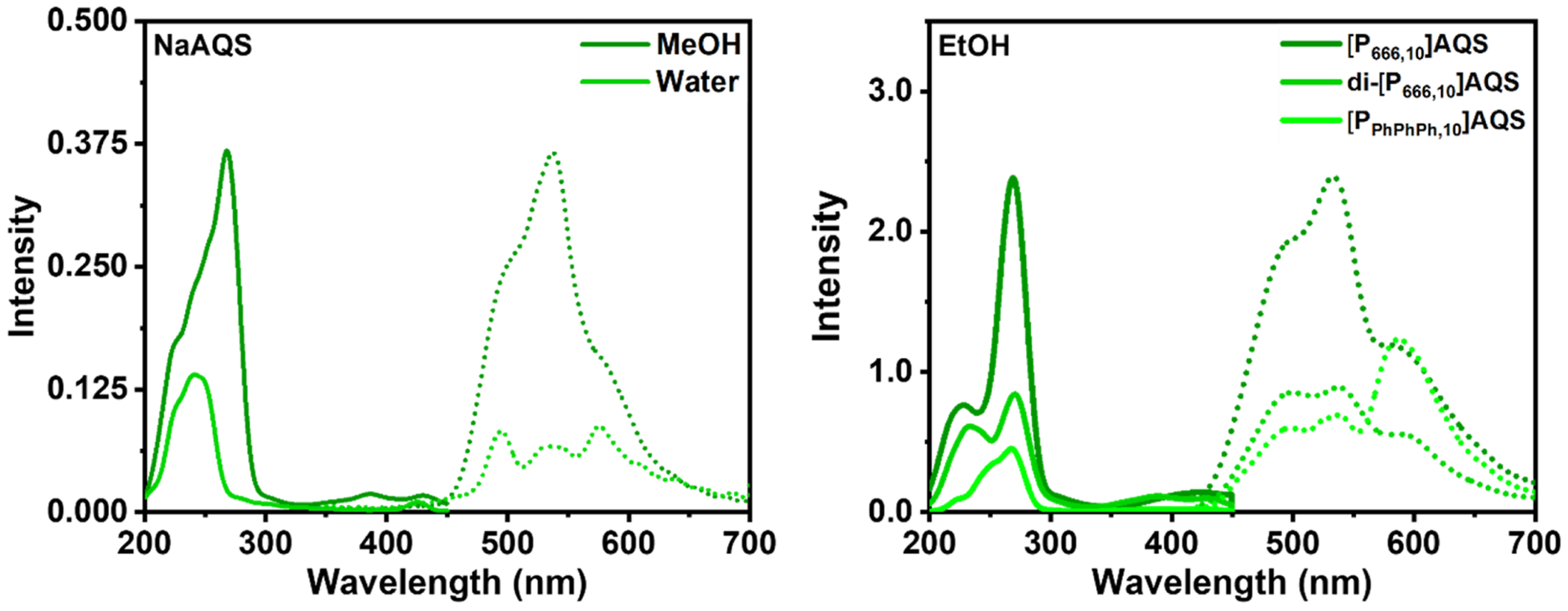
Room-temperature emission/excitation spectra of NaAQS (left) and PILs-AQS in EtOH (right). All spectra were measured at 5.0 × 10^−5^ M concentrations.

The optical properties of NaAQS were only studied in methanol and water due to solubility limitations. NaAQS was soluble in methanol and water up to 1 × 10^−3^ M, but was not soluble in ethanol or any other solvents. PILs-AQS were only studied in ethanol at 1–5 × 10^−5^ M concentrations because their photoluminescent properties were generally weak in all other solvents, despite their good solubility in other solvents (1 × 10^−3^ M). Their molar absorptivities are summarized in Table 3. The UV–Visible spectra for all salts shared similar λ_abs_ values and peak characteristics, with the exception of [P_PhPhPh,10_]AQS displaying a new peak at λ_abs_ = 230 nm. All UV-visible spectra are contained in the supplementary materials (Figures S47–S51). The photoluminescent properties of NaAQS and PILs-AQS were studied at 5 × 10^−5^ M concentration in their respective solvents (Fig. 4). The mechanism of fluorescence for AQS arises from its photochemical reduction into semiquinone and hydroquinones in solution under UV irradiation in the presence of molecular oxygen (Loeff et al., 1984). Interestingly, di-[P_666,10_](AQS)_2_ and [P_PhPhPh,10_]AQS had multiple emission values in the range of λ_em_ = 425–700 nm. NaAQS had a peak emission value of λ_em_ = 538 nm in MeOH and multiple peaks at λ_em_ = 495 nm, 534 nm, and 576 nm in pure water. [P_666,10_]AQS had a single emission peak at λ_em_ = 538 nm in ethanol. Both di-[P_666,10_](AQS)_2_ and [P_PhPhPh,10_]AQS had three emission peaks at λ_em_ = 500 nm, 538 nm, and 589 nm and at λ_em_ = 498 nm, 538 nm, and 587 nm, respectively. Interestingly, the peak emission at λ_em_ = 587 nm for [P_PhPhPh,10_]AQS increased dramatically compared to what is observed in [P_666,10_]AQS and di-[P_666,10_](AQS)_2_. The AQY for all PILs-AQS solutions were low (*Φ* = <0.05), which could be due to the presence of increased flexibility in the semiquinone and hydroquinone products, which may facilitate nonradiative relaxation pathways.

### 3.5. Photoluminescence properties of PILs-DNS in liquid state and glassy state

**Fig. 5.**
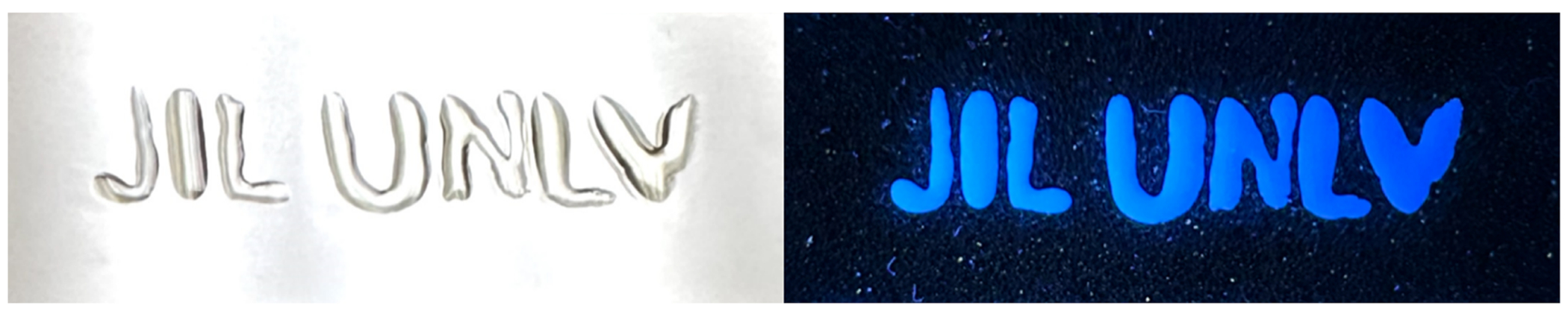
[P_666,10_]DNS in the liquid state on a glass slide under natural light (left) and UV light (right), showcasing bright blue fluorescence.

The AQY of [P_666,10_]DNS and di-[P_666,10_](DNS)_2_ in the liquid state, as well as [P_PhPhPh,10_]DNS in the glassy state, were measured at room temperature. PILs-AQS did not exhibit any appreciable fluorescence in the liquid or glassy state. The fluorescence of [P_666,10_]DNS under UV light is shown in Fig. 5. [P_666,10_]DNS displayed a staggering AQY of 0.35 in the liquid state. The AQY of di-[P_666,10_](DNS)_2_ was also relatively high at 0.26. [P_PhPhPh,10_]DNS in the glassy state possessed an AQY of 0.11, which was the lowest of the three. Typical observation of photoluminescence behavior in pure ionic liquids is generally weak due to aggregation-induced quenching (AIQ). Interestingly, the DNS moiety in the pure liquid or glassy state exhibits aggregation-induced emission (AIE), which is commonly observed in highly luminescent crystals (Xu et al., 2019). Due to the rarity of this phenomenon, there have only been a few LFILs reported in literature with measured AQY values. Trihexyltetradecylphosphonium 8-anilinonaphthalene-1-sulfonate ([P_666,14_] ANS) was reported to have a low AQY of 0.01, despite the similarity in structure to the DNS anion (Delgado et al., 2015). The poor photoluminescence of [P_666,14_]ANS in the liquid state could be attributed to bond rotation of the *N*-phenyl ring of the ANS anion, compared to the highly rigid structure of the DNS anion. Salicylate RTILs with a [P_666,14_] cation were measured to have an AQY of 0.08 while imidazolium salicylate RTILs ([C_2_C_1_Im] and [C_4_C_1_Im]) had AQY values of 0.14 and 0.23, respectively, and pyridinium RTILs ([C_2_C_1_Py] and [C_4_C_1_Py]) had AQY values of 0.04 and 0.03, respectively (Campbell et al., 2014). The selection of cation greatly affects the photoluminescent properties of the ILs in the liquid state due to quenching effects depending on the packing of the liquid from the selection of carbon chain length, the size of cation, aromatic π–π interactions, hydrogen bonding, and other intermolecular forces. Incorporating AFs with PILs is favorable because the tetraalkylphosphonium cation lacks a chromophore and therefore does not quench the fluorescence of the anionic fluorophore. Although they are liquids or glass at RT, their AQYs are surprisingly high due to AIE. Fluorescent glasses with high processability have been synthesized for potential applications in photovoltaic cells, optoelectronic devices, and scintillators for high-resolution X-ray imaging (Ku et al., 2018; Yu et al., 2024).

### 3.6. Photostability of PILs in ethanol

**Fig. 6.**
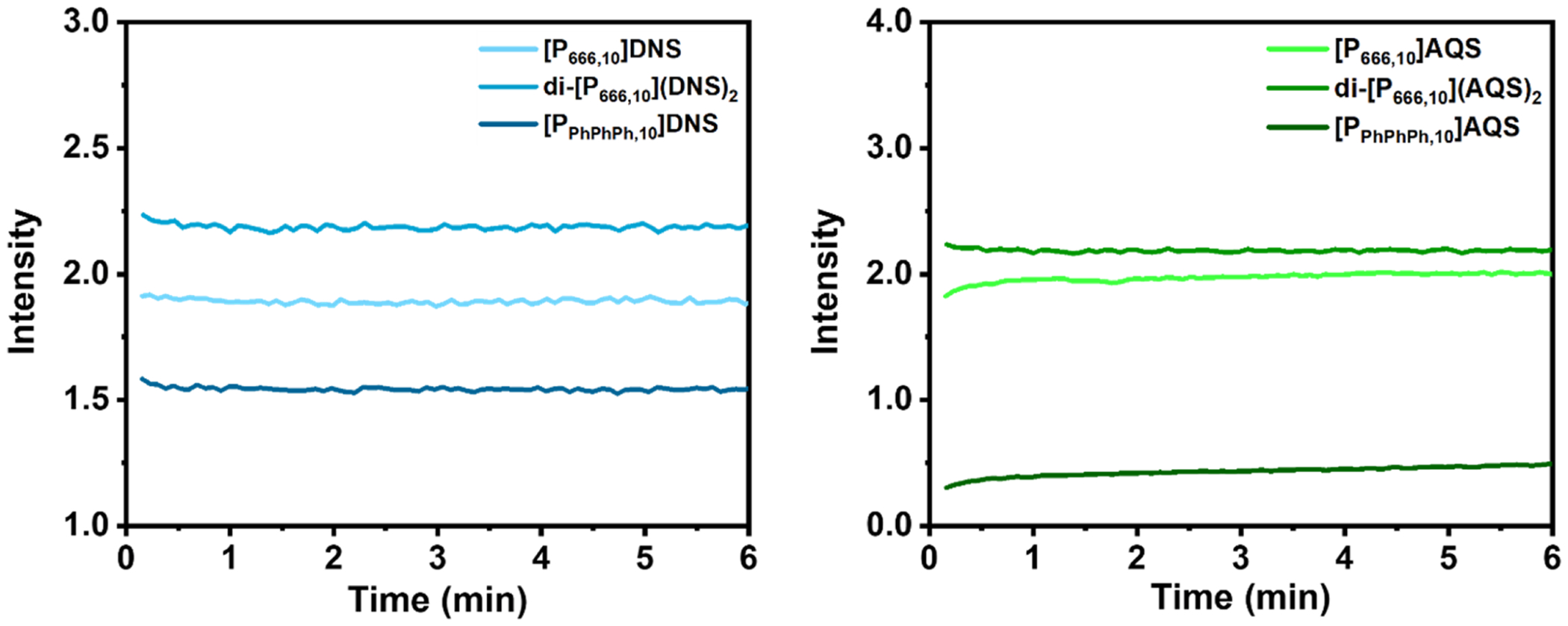
Intensity *vs.* time of PILs-DNS (left) and PILs-AQS (right) in EtOH at 5.0 × 10^−5^ M concentration to measure photobleaching resistance.

The photostability of the fluorophore is an important intrinsic property where emission of constant light occurs multiple times without any chemical degradation. This property makes fluorescence a sensitive technique for visualizing microscopic samples, such as even a small amount of the fluorescent stain can be detected. The photostabilities of all PILs were measured in EtOH at 5.0 × 10−5 M concentration by pulsing the maximum excitation wavelength over the course of 6 min Fig. 6. All PILs demonstrated exceptional photostability, as no significant change in fluorescence intensity was observed during irradiation. This resistance to photobleaching is attributed to the lack of photoreactivity of the functional groups that are present in DNS and AQS.

## 4. Conclusions

A series of PILs-DNS, highly fluorescent in the solution and liquid or glassy state, and fluorescent PILs-AQS were synthesized using an improved synthesis of [P_666,10_]Cl and di-[P_666,10_](Cl)_2_. Their purity was confirmed by ^1^H and ^13^C NMR as well as elemental analysis. Their thermal stabilities were measured by TGA and their phase transitions were determined by DSC, confirming that all PILs were ILs. All [P_666,10_] containing PILs were identified as RTILs and [P_PhPhPh,10_] PILs existed as a glassy state. Their photoluminescent properties were also characterized in various solutions and the liquid or glassy state, with PILs-DNS exhibiting high AQYs in both states and resistance to photobleaching. The PILs-AQS possessed weak photoluminescent properties in solution and no fluorescence in the liquid or glass state. Novel LFILs were successfully synthesized and, in addition to contributing towards the scarcity of these interesting materials in the literature, insights can be drawn to design systems that utilize these novel LFILs for task-specific optoelectronic, sensing, and imaging applications without the need for solvents.

## Supplementary Material

1

Supplementary material associated with this article can be found, in the online version, at doi: 10.1016/j.jil.2025.100156.

## Figures and Tables

**Table 1:** Thermal properties of organic salts and PILs.

Compound	*T*_g_ ( °C)	*T*_m_ ( °C)	*T*_d_ ( °C)
KDNS	–	334	368
NaAQS	–	–	428
[P_666,10_]Cl	–	–	301
di-[P_666,10_](Cl)_2_	–	40	332
[P_666,10_]DNS	–	–	377
di-[P_666,10_](DNS)_2_	−28.81	–	383
[P_PhPhPh,10_]DNS	21.13	–	332
[P_666,10_]AQS	−34.67	–	354
di-[P_666,10_](AQS)_2_	−0.88	107	373
[P_PhPhPh,10_]AQS	30.14	–	359

**Table 2:** Optical absorption and photoluminescent properties of KDNS and PILs-DNS in solutions.

Properties	S	KDNS	[P_666,10_]DNS	di-[P_666,10_](DNS)_2_	[P_PhPhPh,10_]DNS
λ_abs em_ wavelength (nm)	**EtOH**	218, 246, 319	218, 245, 321	218, 245, 325	217, 322
	**MeOH**	217, 246, 319	217, 246, 322	218, 245, 321	218, 320
	**DCM**		246, 325	247, 323	320
	**ACN**	220, 245, 319	220, 245, 321	220, 245, 318	220, 318
	**THF**		237, 306	245, 317	231, 311
	**EtOAc**		314	314	313
	**Toluene**		318	318	318
	**Water**	215, 241, 314			
Molar absorptivity (M^−1^ cm^−1^)	**EtOH**	ε_218_ = 38,811 ± 1633	ε_218_ = 38,712 ± 1194	ε_245_ = 27,454 ± 214	ε_322_ = 4397 ± 177
	**MeOH**	ε_217_ = 38,297 ± 876	ε_217_ = 39,883 ± 1330	ε_245_ = 27,977 ± 266	ε_320_ = 4302 ± 214
	**DCM**		ε_246_ = 15,325 ± 457	ε_247_ = 33,810 ± 189	ε_320_ = 5946 ± 249
	**ACN**	ε_220_ = 38,278 ± 962	ε_220_ = 40,079 ± 719	ε_245_ = 25,550 ± 167	ε_220_ = 34,838 ± 459
	**THF**		ε_237_ = 34,870 ± 2534	ε_245_ = 27,932 ± 352	ε_231_ = 15,564 ± 491
	**EtOAc**		ε_314_ = 5582 ± 84	ε_314_ = 11,409 ± 90	ε_313_ = 5287 ± 198
	**Toluene**		ε_318_ = 5055 ± 77	ε_318_ = 11,159 ± 93	ε_318_ = 5286 ± 179
	**Water**	ε_215_ = 37,334 ± 336			
λ_em_ wavelength (nm)	**EtOH**	454	455	453	453
	**MeOH**	474	474	472	470
	**DCM**		438	442	439
	**ACN**	445	443	442	442
	**THF**		441	439	413
	**EtOAc**		428	438	431
	**Toluene**		420	427	426
	**Water**	510			
Quantum yield (Φ)	**EtOH**	0.84	0.77	0.86	0.95
	**MeOH**	0.73	0.64	0.75	0.84
	**DCM**		0.71	0.92	0.49
	**ACN**	0.67	0.54	0.69	0.79
	**THF**		0.5	0.67	0.09
	**EtOAc**		0.42	0.63	0.05
	**Toluene**		0.47	0.55	0.05
	**Water**	0.56			

* IL in solvents, (**S**): ethanol (EtOH), methanol (MeOH), dichloromethane (DCM), acetonitrile (ACN), tetrahydrofuran (THF), ethyl acetate (EtOAc), toluene, and water.

**Table 3:** Optical absorption and photoluminescent properties of NaAQS and PILs-AQS in solution.

Properties	S	NaAQS	[P_666,10_]AQS	di-[P_666,10_](AQS)_2_	[P_PhPhPh,10_]AQS
λ_abs_ wavelength (nm)	**EtOH**		256, 275, 325	256, 272, 326	230, 256, 275, 327
	**MeOH**	207, 255, 274, 322			
	**Water**	206, 256, 276, 331			
Molar absorptivity (M^−1^ cm^−1^)	**EtOH**		ε_256_ = 49,778 ± 852	ε_272_ = 30,868 ± 221	ε_256_ = 52,735 ± 1283
	**MeOH**	ε_256_ = 49,344 ± 823			
	**Water**	ε_255_ = 51,309 ± 849			
λ_em_ wavelength (nm)	**EtOH**		533	500, 538, 589	498, 538, 587
	**MeOH**	538			
	**Water**	495, 534, 576			

## Data Availability

Data will be made available on request.
