# Supplementary material for: Design and synthesis of phosphonium ionic liquids exhibiting strong fluorescence in various solvents and liquid or glassy state
Source: J Ion Liq. Author manuscript; Available in PMC 2025 Jul 20. (PMC12276853; doi:10.1016/j.jil.2025.100156)
Supplement: 1 [file NIHMS2090463-supplement-1.docx]

# **Supplementary Materials**

Design and synthesis of phosphonium ionic liquids exhibiting strong fluorescence in various solvents and liquid or glassy state

David King^a*^, Yan P. Arnaiz^a^, Hari D. Mandal^b^, Haesook Han^a^, Pradip K. Bhowmik^a^

*^a^ Department of Chemistry and Biochemistry,* University *of Nevada Las Vegas, 4505 S. Maryland Parkway, Box 454003, Las Vegas, NV 89154, USA*

*^b^ Department of Biology and Chemistry, Texas A&M International University, 5201 University Boulevard, Laredo, TX 78041, USA*

| **NMR Spectra:** | |  |  |
| --- | --- | --- | --- |
| ^1^H and ^13^C NMR spectra of KDNS in D_2_O | | Page 3 |  |
| ^1^H and ^13^C NMR spectra of [P_666,10_]Cl in acetone-*d_6_* | | Page 4 |  |
| ^1^H and ^13^C NMR spectra of di-[P_666,10_](Cl)_2_ in acetone-*d*_6_ | | Page 5 |  |
| ^1^H and ^13^C NMR spectra of [P_666,10_]DNS in acetone-*d*_6_ | | Page 6 |  |
| ^1^H and ^13^C NMR spectra of di-[P_666,10_](DNS)_2_ in acetone-*d*_6_ | | Page 7 |  |
| ^1^H and ^13^C NMR spectra of [P_PhPhPh,10_]DNS in acetone-*d*_6_ | | Page 8 |  |
| ^1^H and ^13^C NMR spectra of [P_666,10_]AQS in acetone-*d*_6_ | | Page 9 |  |
| ^1^H and ^13^C NMR spectra of di-[P_666,10_](AQS)_2_ in acetone-*d*_6_ | | | Page 10 |
| ^1^H and ^13^C NMR spectra of [P_PhPhPh,10_]AQS in acetone-*d*_6_ | | | Page 11 |
| Thermal Properties | | |  |
| TGA thermograms: | | |  |
| KDNS and NaAQS | | | Page 12 |
| [P_666,10_]Cl and di-[P_666,10_](Cl)_2_ | | | Page 12 |
| DSC thermograms: | | |  |
| KDNS | | | Page 13 |
| NaAQS | | | Page 13 |
| [P_666,10_]Cl | | | Page 14 |
| di-[P_666,10_](Cl)_2_ | | | Page 14 |
| [P_666,10_]DNS | | | Page 15 |
| di-[P_666,10_](DNS)_2_ | | Page 15 | |
| [P_PhPhPh,10_]DNS | | Page 16 | |
| [P_666,10_]AQS | | Page 16 | |
| di-[P_666,10_](AQS)_2_ | | Page 17 | |
| [P_PhPhPh,10_]AQS | | Page 17 | |
| Optical Properties | |  | |
| UV-Visible Spectra | |  | |
| KDNS | | Pages 18-19 | |
| [P_666,10_]DNS | | Pages 20-23 | |
| di-[P_666,10_](DNS)_2_ | | Pages 23-26 | |
| [P_PhPhPh,10_]DNS | | Pages 27-30 | |
| NaAQS | | Pages 30-31 | |
| [P_666,10_]AQS | | Page 31 | |
| di-[P_666,10_](AQS)_2_ | | Page 32 | |
| [P_PhPhPh,10_]AQS | | | Page 32 |
|  | | |  |

|  |  |
| --- | --- |


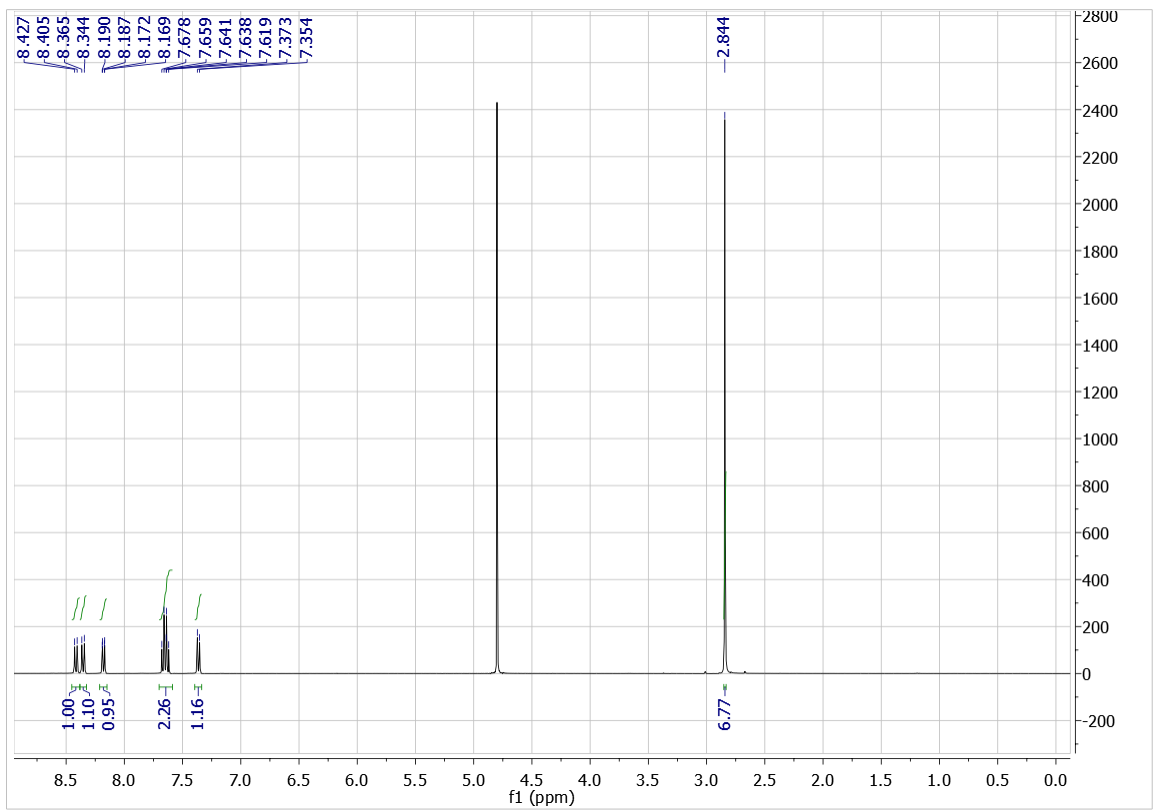


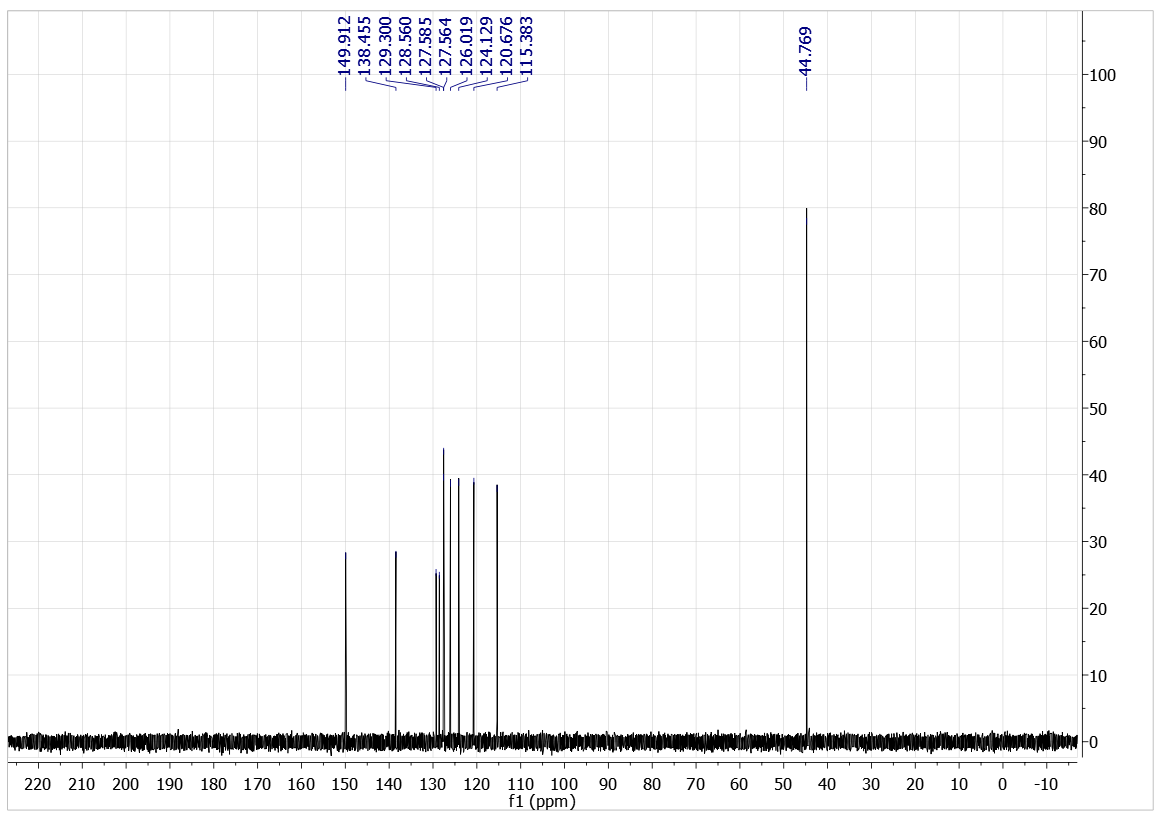


**Figure S1.** ^1^H and ^13^C NMR spectra of KDNS in D_2_O taken at room temperature.


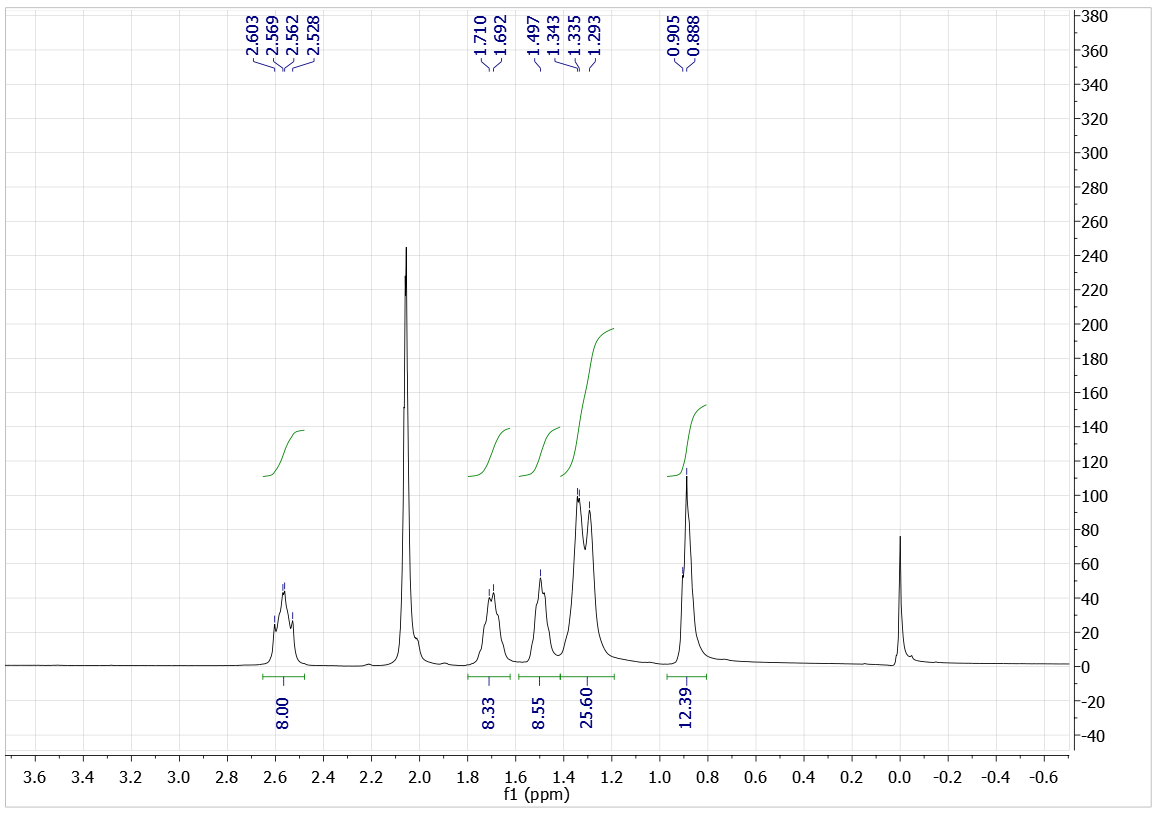

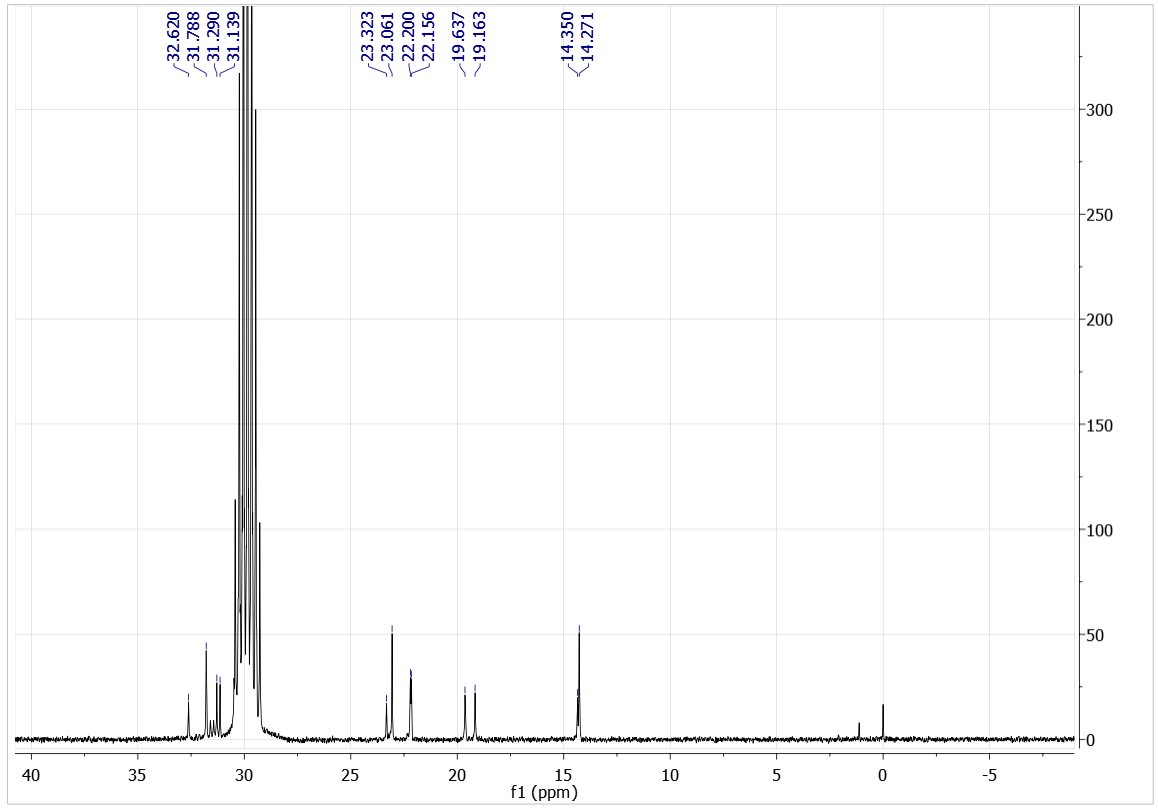


**Figure S2.** ^1^H and ^13^C NMR spectra of [P_666,10_]Cl in acetone-*d*_6_ taken at room temperature.


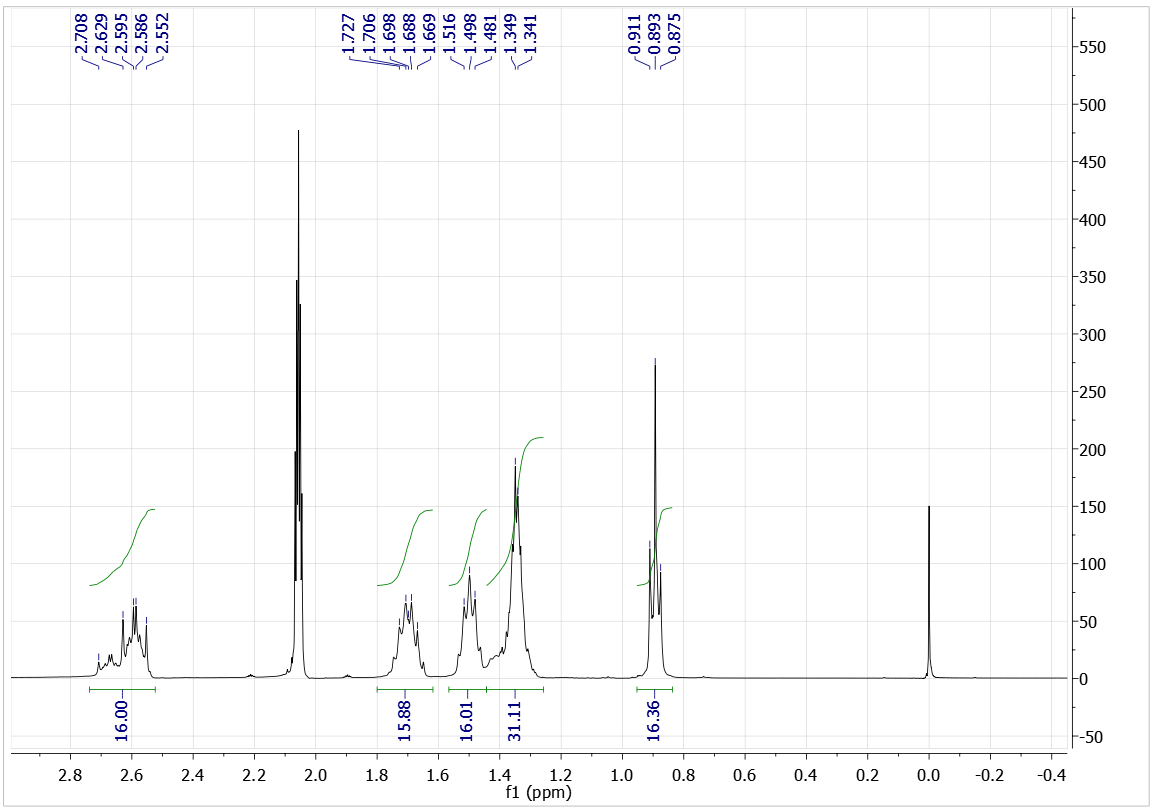

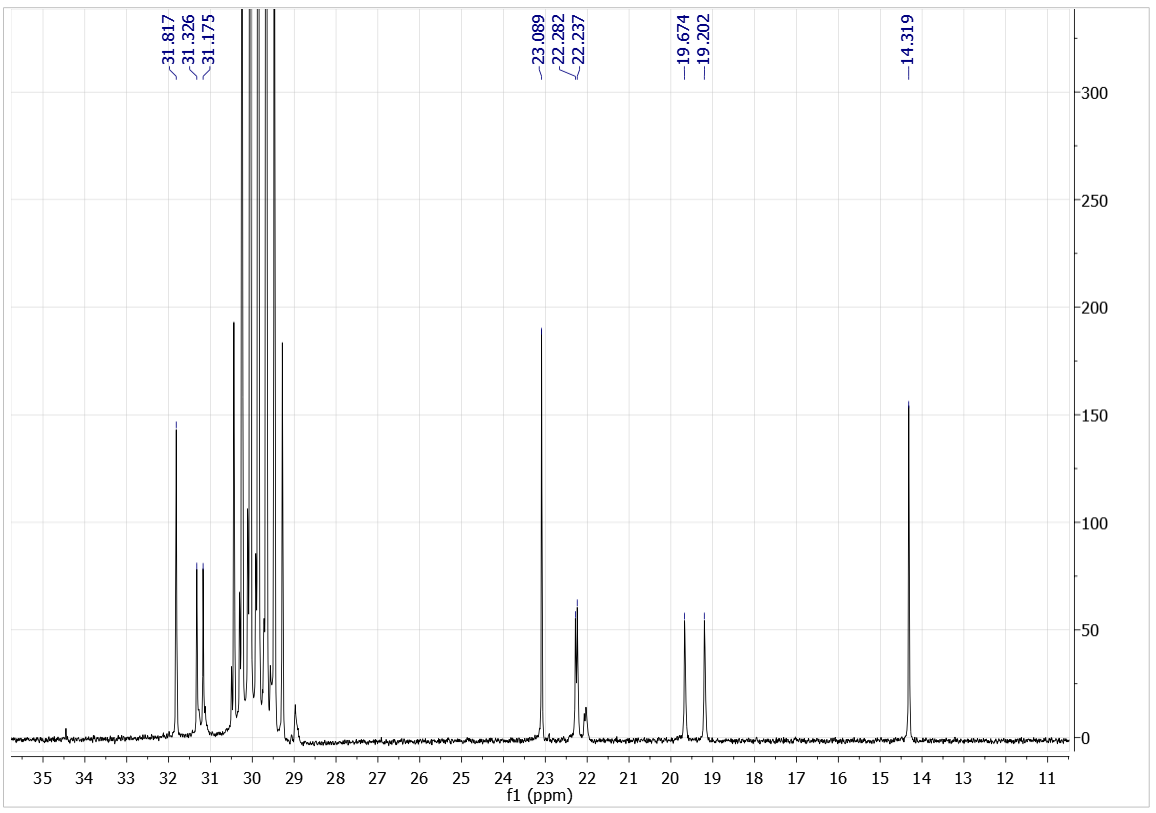


**Figure S3.** ^1^H and ^13^C NMR spectra of di-[P_666,10_](Cl)_2_ in acetone-*d*_6_ taken at room temperature.


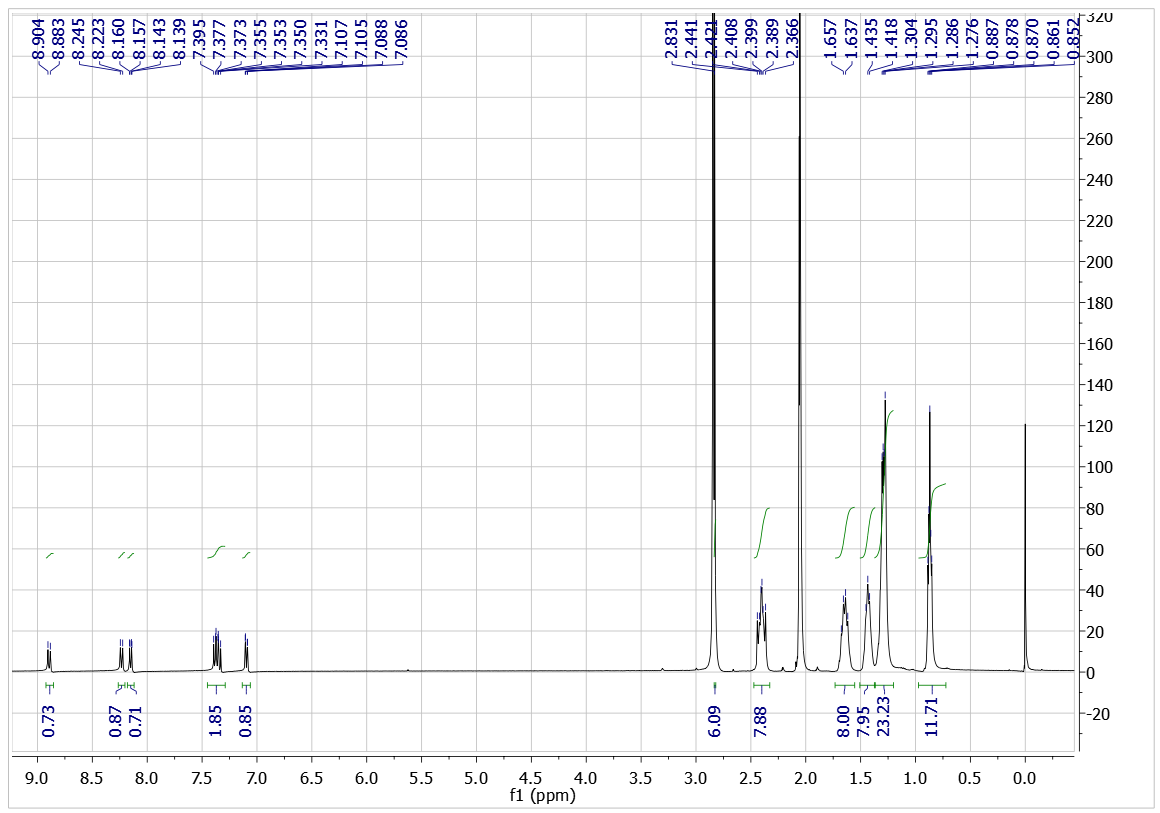


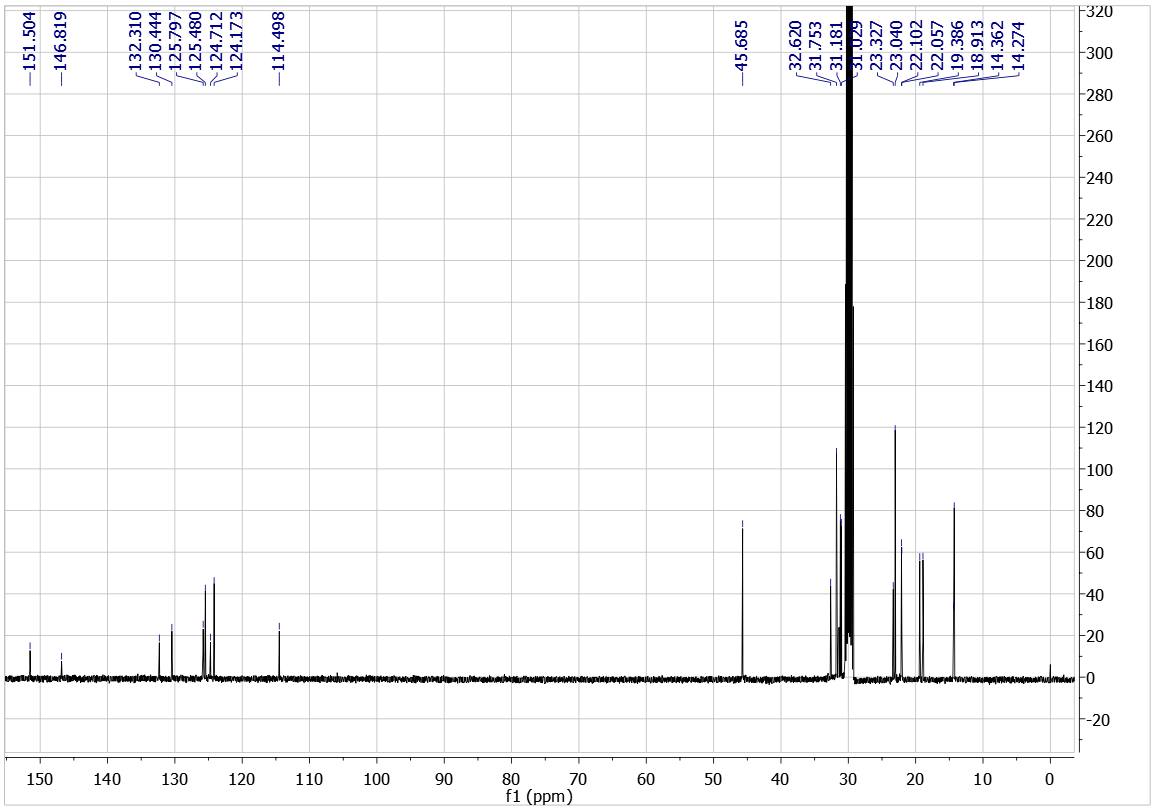


**Figure S4.** ^1^H and ^13^C NMR spectra of [P_666,10_]DNS in acetone-*d*_6_ taken at room temperature.


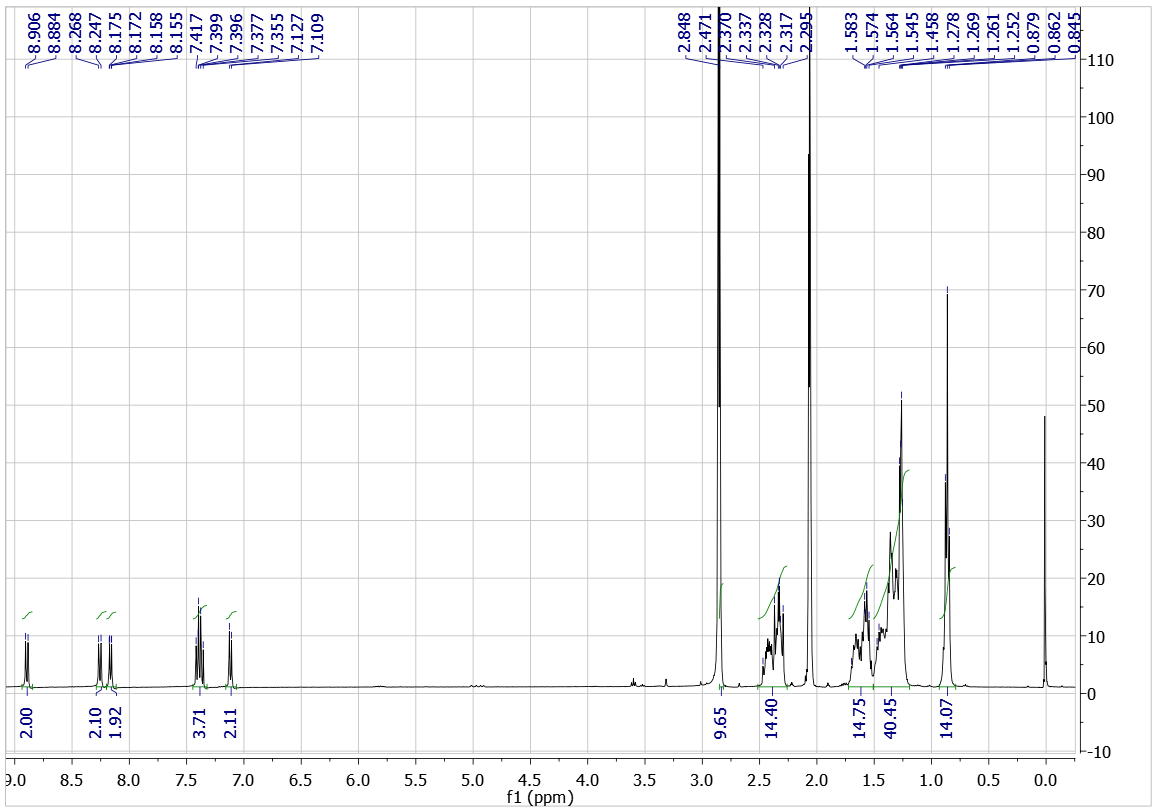


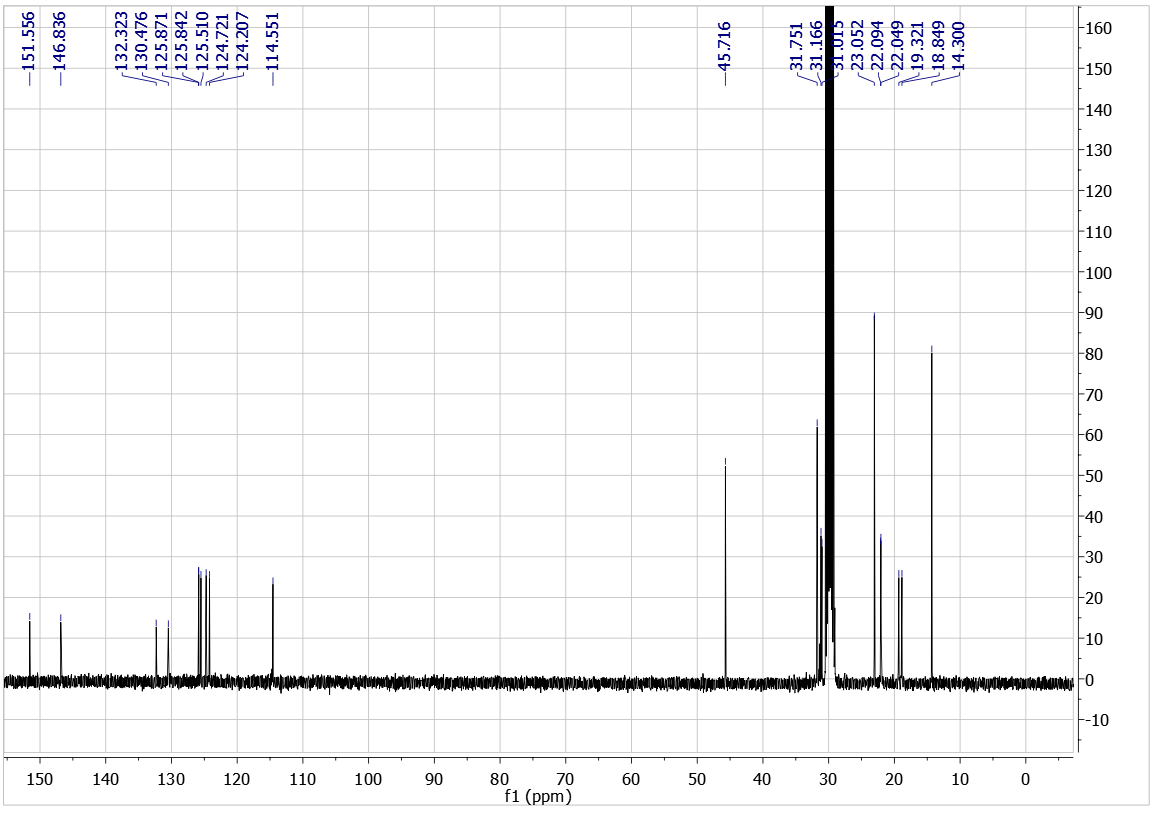


**Figure S5.** ^1^H and ^13^C NMR spectra of di-[P_666,10_](DNS)_2_ in acetone-*d*_6_ taken at room temperature.


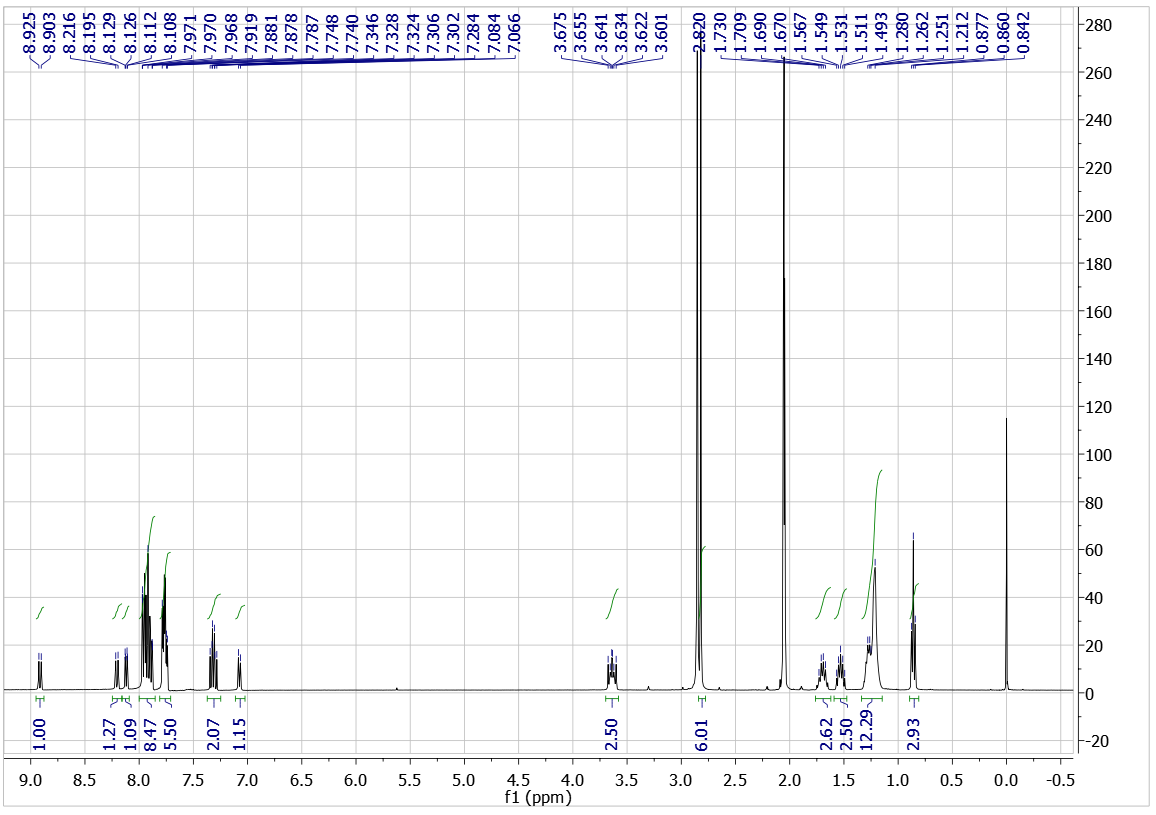

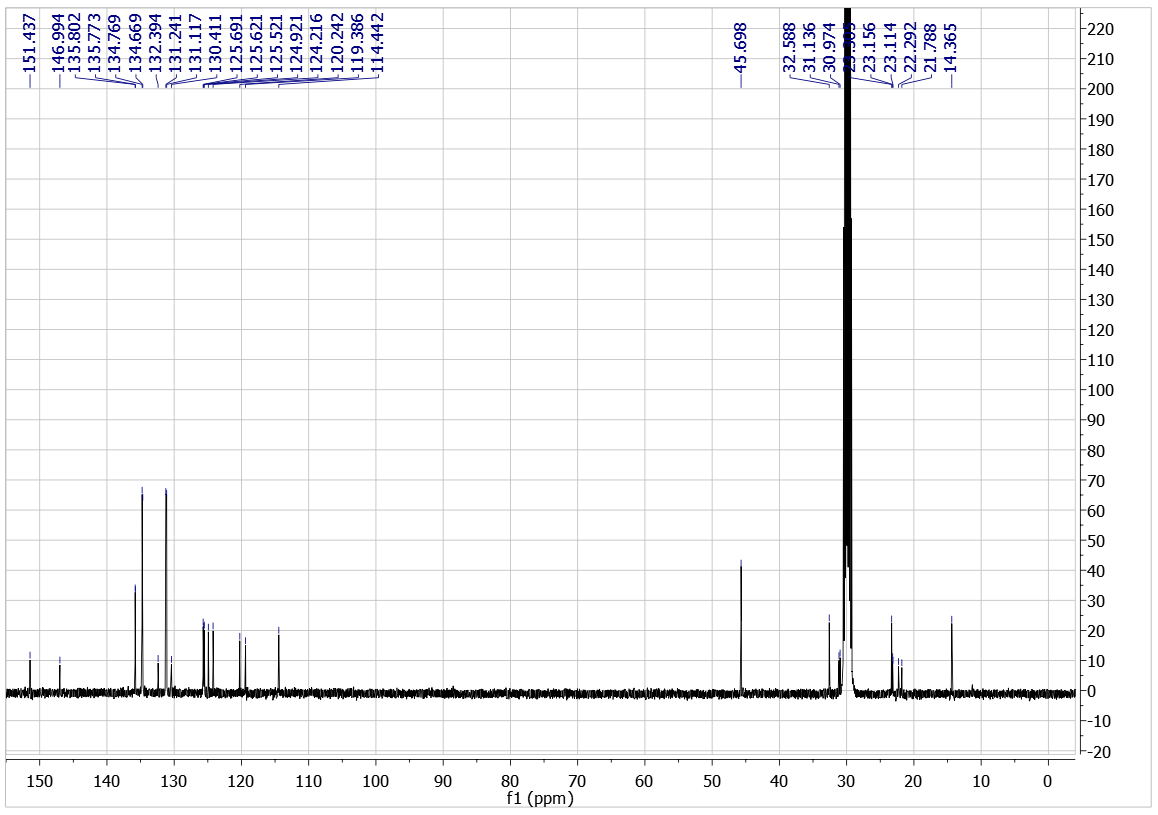


**Figure S6.** ^1^H and ^13^C NMR spectra of [P_PhPhPh,10_]DNS in acetone-*d*_6_ taken at room temperature.


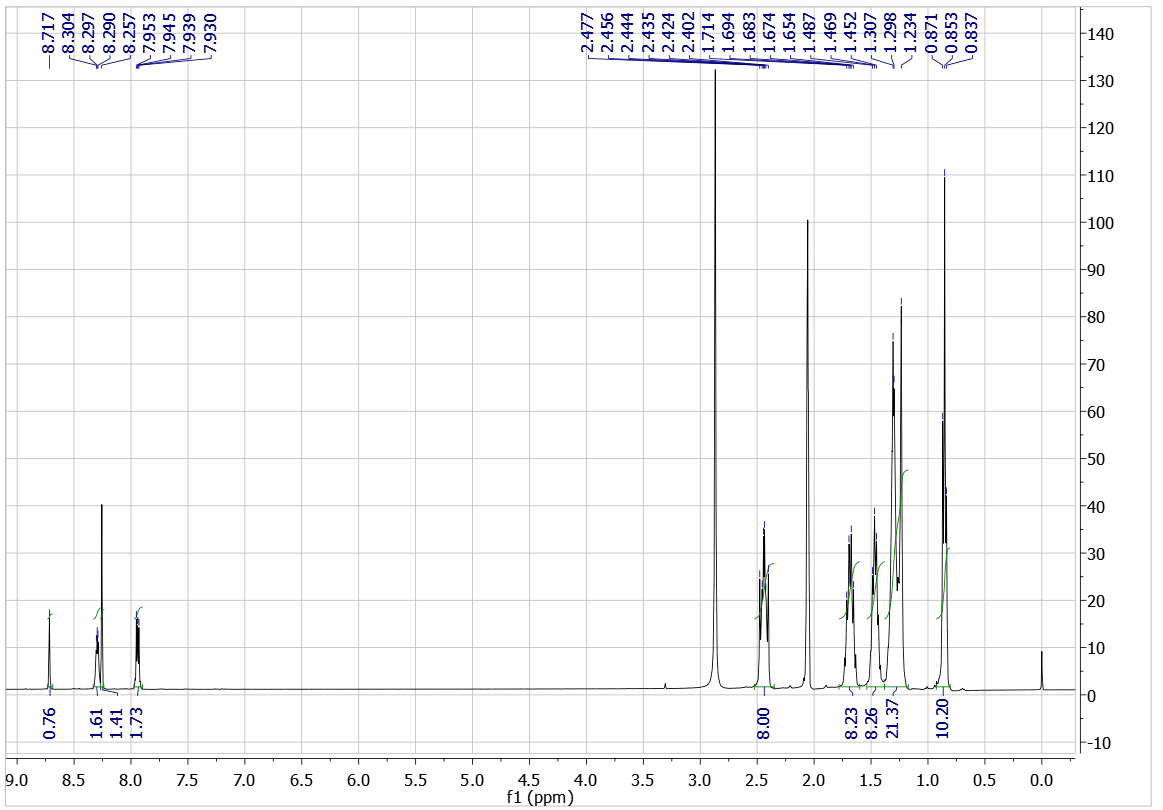


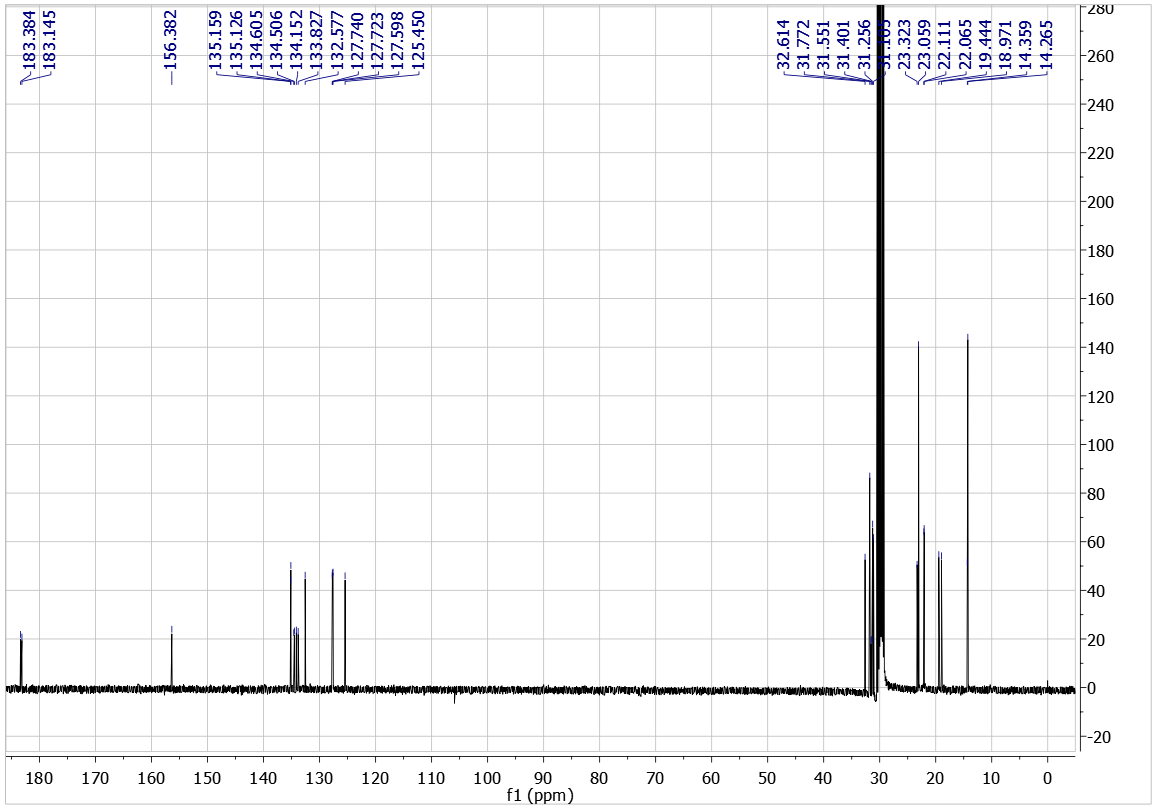


**Figure S7.** ^1^H and ^13^C NMR spectra of [P_666,10_]AQS in acetone-*d*_6_ taken at room temperature.

^
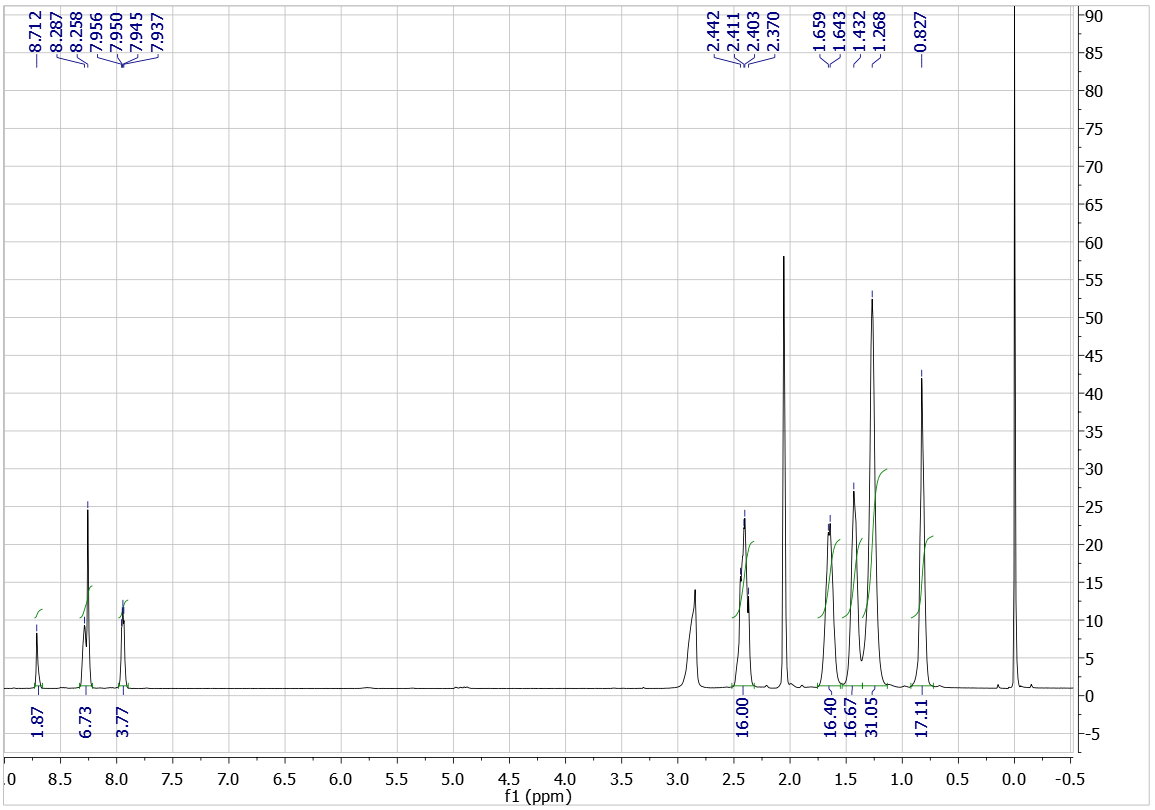
^

^
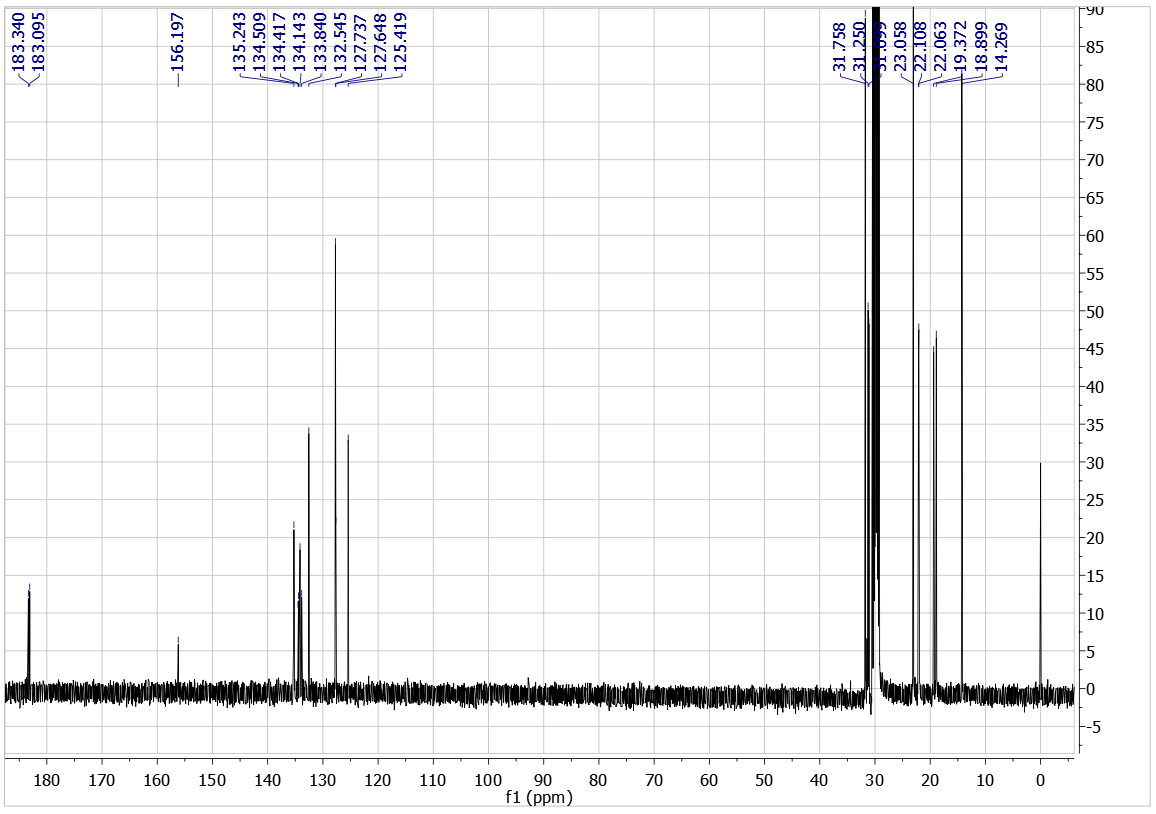
^

**Figure S8.** ^1^H and ^13^C NMR spectra of di-[P_666,10_](AQS)_2_ in acetone-*d*_6_ taken at room temperature.

^
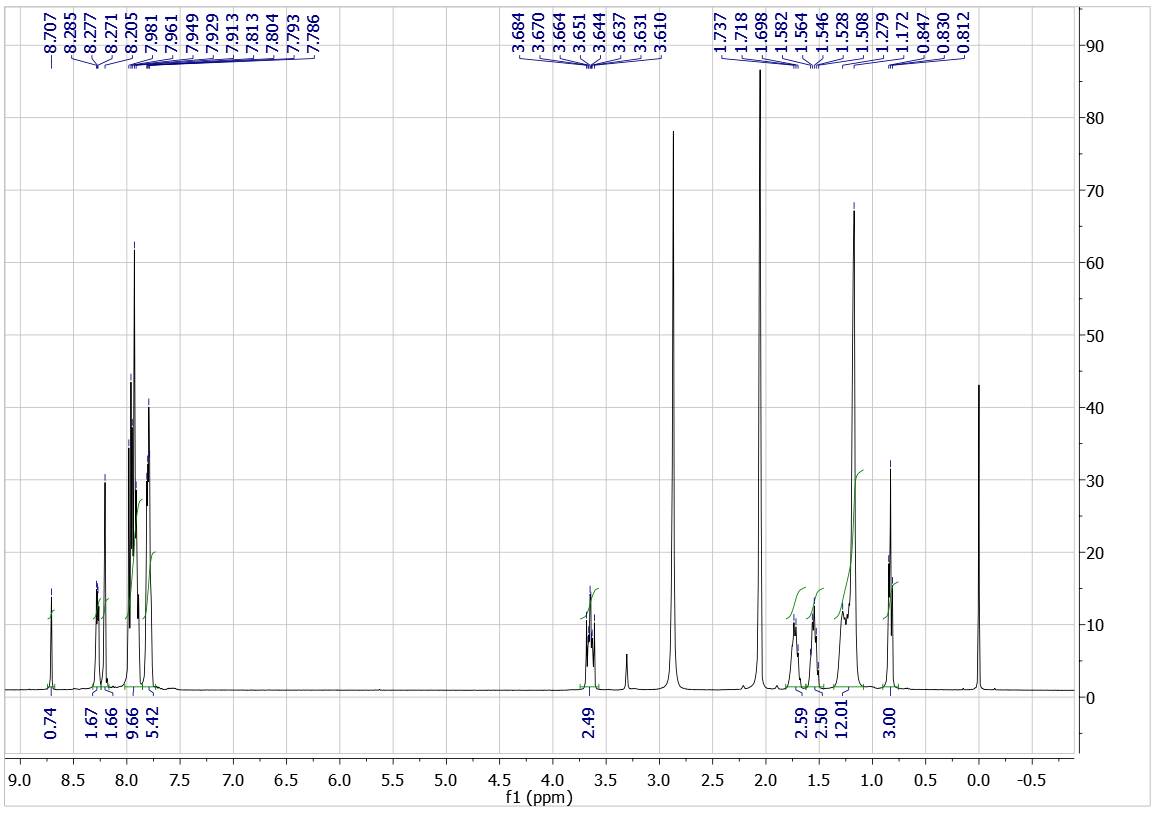

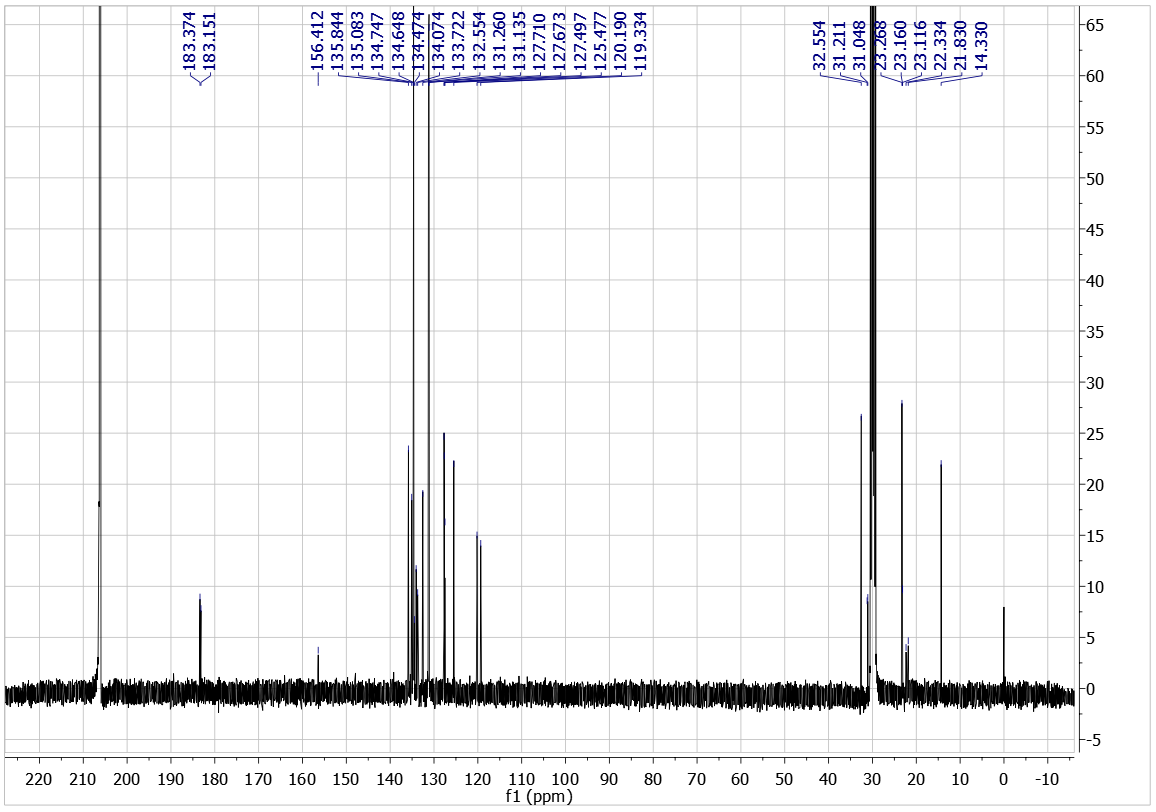
^

**Figure S9.** ^1^H and ^13^C NMR spectra of [P_PhPhPh,10_]AQS in acetone-*d*_6_ taken at room temperature.

**Figure S10.** TGA thermogram of KDNS and NaAQS obtained at a heating rate of 10 ^o^C/min under nitrogen.

**Figure S11.** TGA thermogram of [P_666,10_]Cl and di-[P_666,10_](Cl)_2_ obtained at a heating rate of 10 ^o^C/min under nitrogen.

**Figure S12.** DSC thermogram of KDNS obtained at a heating rate of 10 ^o^C/min under nitrogen.

**Figure S13.** DSC thermogram of NaAQS obtained at a heating rate of 10 ^o^C/min under nitrogen.

**Figure S14.** DSC thermogram of [P_666,10_]Cl obtained at a heating rate of 10 ^o^C/min under nitrogen.

**Figure S15.** DSC thermogram of di-[P_666,10_](Cl)_2_ obtained at a heating rate of 10 ^o^C/min under nitrogen.

**Figure S16.** DSC thermogram of [P_666,10_]DNS obtained at a heating rate of 10 ^o^C/min under nitrogen.

**Figure S17.** DSC thermogram of di-[P_666,10_](DNS)_2_ obtained at a heating rate of 10 ^o^C/min under nitrogen.

**Figure S18.** DSC thermogram of [P_PhPhPh,10_]DNS obtained at a heating rate of 10 ^o^C/min under nitrogen.

**Figure S19.** DSC thermogram of [P_666,10_]AQS obtained at a heating rate of 10 ^o^C/min under nitrogen.

**Figure S20.** DSC thermogram of di-[P_666,10_](AQS)_2_ obtained at a heating rate of 10 ^o^C/min under nitrogen.

**Figure S21.** DSC thermogram of [P_PhPhPh,10_]AQS obtained at a heating rate of 10 ^o^C/min under nitrogen.

ε_218_**-** 38811 ±1633 ε_246_**-** 13613 ±167 ε_319_**-** 4727 ±88

**Figure S22.** UV-visible absorption spectrum and molar absorptivities of KDNS in EtOH.

ε_217_**-** 38297 ±876 ε_246_**-** 12476 ±207 ε_319_**-** 4787 ±161

**Figure S23.** UV-visible absorption spectrum and molar absorptivities of KDNS in MeOH.

ε_220_**-** 38278 ±962 ε_245_**-** 11735 ±186 ε_319_**-** 4718 ±80

**Figure S24.** UV-visible absorption spectrum and molar absorptivities of KDNS in ACN.

ε_215_**-** 37334 ±336 ε_241_**-** 14760 ±301 ε_314_**-** 4527 ±147

**Figure S25.** UV-visible absorption spectrum and molar absorptivities of KDNS in water.

ε_218_**-** 38712 ±1194 ε_245_**-** 13420 ±118 ε_312_**-** 4576 ±102

**Figure S26.** UV-visible absorption spectrum and molar absorptivities of [P_666,10_]DNS in EtOH.

ε_217_**-** 39883 ±1330 ε_246_**-** 13362 ±196 ε_322_**-** 4352 ±142

**Figure S27.** UV-visible absorption spectrum and molar absorptivities of [P_666,10_]DNS in MeOH.

ε_246_**-** 15325 ±457 ε_325_**-** 5325 ±176

**Figure S28.** UV-visible absorption spectrum and molar absorptivities of [P_666,10_]DNS in DCM.

ε_220_**-** 40079 ±719 ε_245_**-** 13065 ±287 ε_321_**-** 5005 ±163

**Figure S29.** UV-visible absorption spectrum and molar absorptivities of [P_666,10_]DNS in ACN.

ε_237_**-** 34870 ±2534 ε_306_**-** 5293 ±94

**Figure S30.** UV-visible absorption spectrum and molar absorptivities of [P_666,10_]DNS in THF.


ε_314_**-** 5582 ±84

**Figure S31.** UV-visible absorption spectrum and molar absorptivities of [P_666,10_]DNS in EtOAc.

ε_318_**-** 5055 ±77

**Figure S32.** UV-visible absorption spectrum and molar absorptivities of [P_666,10_]DNS in toluene.

ε_245_**-** 27454 ±214 ε_325_**-** 9464 ±105

**Figure S33.** UV-visible absorption spectrum and molar absorptivities of di-[P_666,10_](DNS)_2_ in EtOH.

ε_245_**-** 27977 ±266 ε_321_**-** 9827 ±167

**Figure S34.** UV-visible absorption spectrum and molar absorptivities of di-[P_666,10_](DNS)_2_ in MeOH.

ε_247_**-** 33810 ±189 ε_323_**-** 12693 ±111

**Figure S35.** UV-visible absorption spectrum and molar absorptivities of di-[P_666,10_](DNS)_2_ in DCM.

ε_245_**-** 25550 ±167 ε_318_**-** 10434 ±104

**Figure S36.** UV-visible absorption spectrum and molar absorptivities of di-[P_666,10_](DNS)_2_ in ACN.

ε_245_**-** 27932 ±352 ε_317_**-** 11151 ±173

**Figure S37.** UV-visible absorption spectrum and molar absorptivities of di-[P_666,10_](DNS)_2_ in THF.

ε_314_**-** 11409 ±90

**Figure S38.** UV-visible absorption spectrum and molar absorptivities of di-[P_666,10_](DNS)_2_ in EtOAc.

ε_318_**-** 11159 ±93

**Figure S39.** UV-visible absorption spectrum and molar absorptivities of di-[P_666,10_](DNS)_2_ in toluene.

ε_322_**-** 4397 ±177

**Figure S40.** UV-visible absorption spectrum and molar absorptivities of [P_PhPhPh,10_]DNS in EtOH.

ε_320_**-** 4302 ±214

**Figure S41.** UV-visible absorption spectrum and molar absorptivities of [P_PhPhPh,10_]DNS in MeOH.

ε_320_**-** 5946 ±249

**Figure S42.** UV-visible absorption spectrum and molar absorptivities of [P_PhPhPh,10_]DNS in DCM.

ε_220_**-** 34838 ±459 ε_318_**-** 2916 ±207

**Figure S43.** UV-visible absorption spectrum and molar absorptivities of [P_PhPhPh,10_]DNS in ACN.

ε_231_**-** 15564 ±491 ε_311_**-** 3001 ±228

**Figure S44.** UV-visible absorption spectrum and molar absorptivities of [P_PhPhPh,10_]DNS in THF.

ε_313_**-** 5287 ±198

**Figure S45.** UV-visible absorption spectrum and molar absorptivities of [P_PhPhPh,10_]DNS in EtOAc.

ε_218_**-** 5286 ±179

**Figure S46.** UV-visible absorption spectrum and molar absorptivities of [P_PhPhPh,10_]DNS in toluene.

ε_207_**-** 33050 ±1081 ε_255_**-** 51309 ±849 ε_274_**-** 16307 ±328 ε_322_**-** 5627 ±166

**Figure S47.** UV-visible absorption spectrum and molar absorptivities of NaAQS in MeOH.

ε_206_**-** 32601 ±559 ε_256_**-** 49344 ±823 ε_276_**-** 15296 ±396 ε_331_**-** 5573 ±140

**Figure S48.** UV-visible absorption spectrum and molar absorptivities of NaAQS in water.

ε_256_**-** 49778 ±852 ε_275_**-** 15417 ±344 ε_325_**-** 5451 ±90

**Figure S49.** UV-visible absorption spectrum and molar absorptivities of [P_666,10_]AQS in EtOH.

ε_272_**-** 30868 ±221 ε_326_**-** 11379 ±117

**Figure S50.** UV-visible absorption spectrum and molar absorptivities of di-[P_666,10_](AQS)_2_ in EtOH.

ε_230_**-** 36158 ±324 ε_256_**-** 52735 ±1283 ε_275_**-** 19746 ±172 ε_327_**-** 5701 ±132

**Figure S51.** UV-visible absorption spectrum and molar absorptivities of [P_PhPhPh,10_]AQS in EtOH.
